# The Physiology and Pathophysiology of T-Tubules in the Heart

**DOI:** 10.3389/fphys.2021.718404

**Published:** 2021-09-09

**Authors:** Ingunn E. Setterberg, Christopher Le, Michael Frisk, Harmonie Perdreau-Dahl, Jia Li, William E. Louch

**Affiliations:** ^1^Institute for Experimental Medical Research, Oslo University Hospital and University of Oslo, Oslo, Norway; ^2^KG Jebsen Centre for Cardiac Research, University of Oslo, Oslo, Norway

**Keywords:** t-tubules, dyad, cardiomyocyte, calcium homeostasis, heart failure

## Abstract

In cardiomyocytes, invaginations of the sarcolemmal membrane called t-tubules are critically important for triggering contraction by excitation-contraction (EC) coupling. These structures form functional junctions with the sarcoplasmic reticulum (SR), and thereby enable close contact between L-type Ca^2+^ channels (LTCCs) and Ryanodine Receptors (RyRs). This arrangement in turn ensures efficient triggering of Ca^2+^ release, and contraction. While new data indicate that t-tubules are capable of exhibiting compensatory remodeling, they are also widely reported to be structurally and functionally compromised during disease, resulting in disrupted Ca^2+^ homeostasis, impaired systolic and/or diastolic function, and arrhythmogenesis. This review summarizes these findings, while highlighting an emerging appreciation of the distinct roles of t-tubules in the pathophysiology of heart failure with reduced and preserved ejection fraction (HFrEF and HFpEF). In this context, we review current understanding of the processes underlying t-tubule growth, maintenance, and degradation, underscoring the involvement of a variety of regulatory proteins, including junctophilin-2 (JPH2), amphiphysin-2 (BIN1), caveolin-3 (Cav3), and newer candidate proteins. Upstream regulation of t-tubule structure/function by cardiac workload and specifically ventricular wall stress is also discussed, alongside perspectives for novel strategies which may therapeutically target these mechanisms.

## T-Tubule Structure and Function

Forceful contraction of the heart requires coordinated contraction of cardiac muscle cells, called cardiomyocytes. Within these cells, electrical excitation during the action potential is linked to cell shortening by a process known as excitation-contraction (EC) coupling. In mammalian heart, this process is made possible by membrane invaginations called t-tubules which propagate the action potential into the cell interior. Critical in normal cardiac physiology and importantly disrupted during disease, t-tubules have been the focus of considerable research focus in the past decades. This review will summarize this progress and outline future perspectives, with particular attention given to potential roles of t-tubules as therapeutic targets.

### Structural Overview

Retzius (1881) first suggested t-tubules’ existence in 1881 after he noted that muscle cells exhibit a quick inward spread of electrical depolarization (“negative variation”). Thereafter, the first visual evidence of t-tubules was provided by Nyström (1897), who found that India ink entered rabbit heart muscle in a transverse pattern. In 1957, with the help of electron microscopy, Lindner (1957) clearly described t-tubule structures in dog ventricular cardiomyocytes. Subsequent studies from both skeletal and cardiac muscle revealed that t-tubules serve to rapidly conduct electrical excitation and facilitate communication with the sarcoplasmic reticulum (SR) (Huxley and Taylor, 1955; Cheng et al., 1996), thereby playing a central role in EC coupling. More recently, the advent of newer techniques, such as three-dimensional (3D) electron microscopy, super-resolution microscopy, and ion-conductance microscopy have provided ever greater detail in current understanding of t-tubule biology (Gu et al., 2002; Hayashi et al., 2009; Pinali et al., 2013; Crossman et al., 2017; Frisk et al., 2021).

**FIGURE 1:**
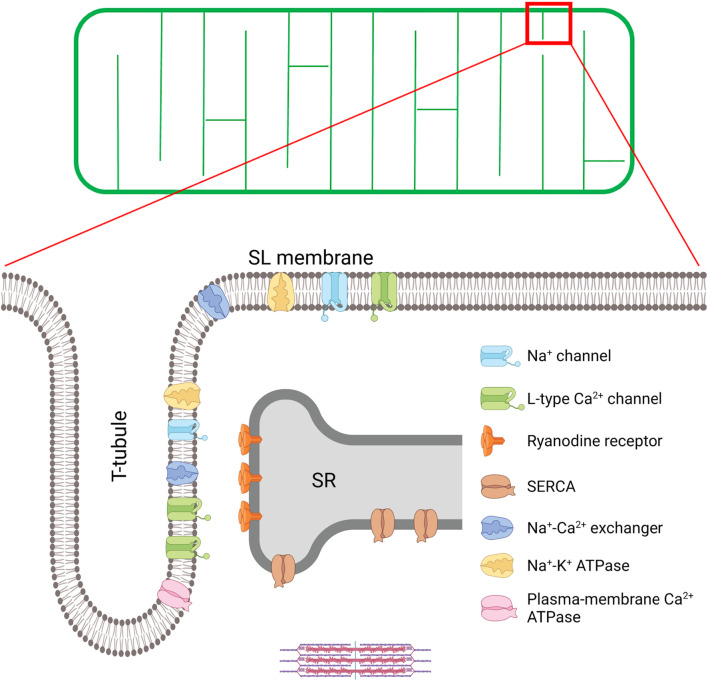
T-tubule structure and key proteins involved in excitation-contraction (EC) coupling in the cardiomyocyte. A schematic overview of the t-tubule network is provided in the upper panel, while an enlargement of the indicated region is provided below to illustrate positioning of EC coupling proteins. EC coupling is initiated as Na^+^ channels are opened, and the cell membrane depolarizes during the action potential. This depolarization triggers the opening of voltage-gated L-type Ca^2+^-channels (LTCCs) in the t-tubules, and subsequent Ca^2+^-induced Ca^2+^ release from the SR through the opening of Ryanodine Receptors (RyRs). This process occurs at specialized junctions called dyads, where LTCCs and RyRs are in close proximity. After released Ca^2+^ binds to the myofilaments to trigger contraction, Ca^2+^ is recycled into the SR by the sarco-endoplasmic reticulum ATPase (SERCA), and removed from the cell by the Na^+^-Ca^2+^ exchanger (NCX) and the plasma-membrane Ca^2+^ ATPase. NCX activity is critically regulated by Na^+^ levels, set by the Na^+^ channel and the Na^+^-K^+^ ATPase within t-tubules. Created with BioRender.com.

A schematic overview of cardiomyocyte t-tubule structure is provided in Figure 1. Originally named transverse tubules, the majority of these structures are indeed oriented transversely across the cell, in a well-organized network along the Z-lines (Lindner, 1957; McNutt, 1975; Kostin et al., 1998). However, a fraction of tubules is positioned along the cardiomyocyte’s longitudinal axis, extending into the A-band of the sarcomere (Simpson, 1965; Forssmann and Girardier, 1970; Sperelakis and Rubio, 1971; Forbes and Sperelakis, 1976; Soeller and Cannell, 1999; Pinali et al., 2013). In an effort to accurately describe this arrangement, and include both populations of t-tubules, some have referred to the overall network as the transverse-axial tubule system (TATS) (Sperelakis and Rubio, 1971; Forbes and Sperelakis, 1976). In rat cardiomyocytes, the proportions of transverse and longitudinal t-tubules are estimated at 60 and 40%, respectively (Soeller and Cannell, 1999), although as noted below there are large species differences. In addition to variation in overall orientation, t-tubules also exhibit non-uniform branching, tortuosity, bulges, and folds (Jayasinghe et al., 2012; Hong et al., 2014), and the lumen diameter of the t-tubules varies from 20 to 450 nm (Soeller and Cannell, 1999). These narrow dimensions of the t-tubules are believed to restrict solute movement compared to the extracellular environment, with important physiological consequences (Swift et al., 2006; Kong et al., 2018b; Uchida and Lopatin, 2018). However, a recent publication demonstrated that there is significant deformation of t-tubules throughout the cardiomyocyte contractile cycle. During contraction, extracellular solution is thus actively pumped in and out of the t-tubular compartment in a rate-dependent manner, counteracting geometrical diffusion constraints, and representing a new form of t-tubule functional autoregulation which has been hitherto underappreciated (Rog-Zielinska et al., 2021b).

There are considerable species differences in t-tubule density and organization (Soeller and Cannell, 1999). Indeed, depending on species, t-tubules constitute 0.8–3.6% of the total cell volume, and 21–64% of the total sarcolemma (Stewart and Page, 1978; Severs et al., 1985; Bers, 2002; Hayashi et al., 2009). In smaller species with higher heart rates (mouse and rat), t-tubule arrangement is the most complex, with denser, more branched and thinner structures than large mammals (rabbit, pig, and human) (Soeller and Cannell, 1999; Cannell et al., 2006; Savio-Galimberti et al., 2008; Jayasinghe et al., 2012; Kong et al., 2018b). Mouse cardiomyocytes also exhibit smaller t-tubule openings, and thus it has been proposed that t-tubular solute movement is more restricted in these cells than in larger species (Kong et al., 2018b). T-tubule topology also varies across the different heart chambers. While t-tubule structure is reported to be similarly well-organized in both left and right ventricle (Tidball et al., 1991; Chen et al., 2013; Guo et al., 2013), there is greater variance reported in the atria. Although t-tubules have been identified in atrial myocytes in both small (rat) (Frisk et al., 2014) and larger mammals (pig, dog, sheep, cow, horse, and human) (Dibb et al., 2009; Lenaerts et al., 2009; Richards et al., 2011; Frisk et al., 2014; Arora et al., 2017), studies in rat and pig indicated that only a minority of atrial cells contain tubular structures (Frisk et al., 2014; Gadeberg et al., 2016). When they are present in atrial cells, t-tubules are considerably less developed than those present in ventricular myocytes, with generally lower density and often a more longitudinal orientation (Dibb et al., 2009; Lenaerts et al., 2009; Smyrnias et al., 2010; Richards et al., 2011; Frisk et al., 2014; Glukhov et al., 2015; Gadeberg et al., 2016; Arora et al., 2017). Notably, t-tubule density is reported to be higher in the atria’s epicardium than in the endocardium (Frisk et al., 2014). Although not yet closely examined, there may also be intra-chamber variability in t-tubule structure, as lower t-tubule density has been reported in the apex than other regions of the left ventricle (Wright et al., 2018; Frisk et al., 2021).

### Functional Overview

As central players in EC-coupling, t-tubule structure is closely linked to contractile function. T-tubules carry the action potential (AP) deep into the cell interior, and the resulting depolarization of the t-tubular membrane leads to opening of voltage-gated L-type Ca^2+^ channels (LTCCs, Ca_v_1.2; see Figure 1). This influx of Ca^2+^ from the extracellular space triggers a larger amount of Ca^2+^ release from the SR through ryanodine receptors (RyRs); a process called Ca^2+^-induced Ca^2+^ release (CICR) (Fabiato, 1983; Bers, 2002). Binding of the released Ca^2+^ to the myofilaments results in the contraction of the cell, and thus the whole heart. Thereafter, relaxation occurs as Ca^2+^ is recycled into the SR by the SR Ca^2+^-ATPase (SERCA), or extruded from the cell by the Na^+^/Ca^2+^ exchanger (NCX), and to a lesser extent, the sarcolemmal Ca^2+^ ATPase (Louch et al., 2012).

The presence of a dense and well-organized t-tubule network ensures synchronous Ca^2+^ release across the cell. This is particularly apparent in mouse and rat ventricular cardiomyocytes, due to the robust presence of these structures (Heinzel et al., 2002; Louch et al., 2006; Song et al., 2006). However, in larger animals, such as pig, less synchronous Ca^2+^ release has been linked to the lower cardiomyocyte t-tubule density (Heinzel et al., 2002, 2008). Similarly, in atrial cells with a less developed t-tubule network, the Ca^2+^ transient is less synchronous, with a wave-like propagation of released Ca^2+^ from the periphery toward the cell interior (Frisk et al., 2014). This dyssynchronous pattern of Ca^2+^ release can be reproduced by experimentally detubulating cardiomyocytes (Louch et al., 2004; Brette et al., 2005).

Triggering of Ca^2+^ release from the SR requires its close alignment with the t-tubule membrane at “dyads” (Figure 1), and close proximity between LTCCs and RyRs (Sun et al., 1995). While EM data have historically indicated that the dyadic cleft measures only ≈12 nm across (Takeshima et al., 2000), recent data have suggested that the true dimensions may be even more narrow (<10 nm), and that artifactual expansion of the cleft could have occurred in earlier work as a result of sample fixation procedures (Rog-Zielinska et al., 2021a). Consistent dyadic dimensions are ensured by junctophilin-2 (JPH2) which stabilizes the membranes, but also functionally interacts with both RyRs and LTCCs (Jiang et al., 2016; Munro et al., 2016; Reynolds et al., 2016; Gross et al., 2021). Insight into the precise positioning of these dyadic proteins has been made possible by new advances in super-resolution microscopy, including STED (Wagner et al., 2012), 3d STORM (Shen et al., 2019), and DNA-PAINT techniques (Jayasinghe et al., 2018; Sheard et al., 2019). For example, nanoscale visualization of RyRs has shown that these proteins are organized into clusters containing an average of 9–14 channels (Baddeley et al., 2009; Jayasinghe et al., 2018; Shen et al., 2019). Tight packing of RyRs within these clusters is thought to synchronize their gating (Marx et al., 2001; Sobie et al., 2006). At the larger scale, RyR clusters that are in close proximity have been predicted to cooperate as a Ca^2+^ release unit (CRU), or a “super-cluster,” with released Ca^2+^ jumping between nearby clusters to produce a Ca^2+^ spark (Sobie et al., 2006; Baddeley et al., 2009; Louch et al., 2013; Kolstad et al., 2018). CRUs are estimated to contain an average of between 18 and 23 RyRs (Shen et al., 2019), and this composition is a key factor in determining Ca^2+^ spark frequency and amplitude (Xie et al., 2019).

On the t-tubule side of the dyad, LTCCs are localized opposite RyR clusters. LTCCs also form clusters, with sizes estimated to be about 67% of those reported for RyRs (Scriven et al., 2010). Exciting new data indicate that the interaction between LTCCs facilitates Ca^2+^ influx *via* coupled gating of the channels (Dixon et al., 2015). Recently, Ito and colleagues found that β-adrenergic receptor activation augments LTCC abundance and promotes enhanced channel interaction, thereby amplifying Ca^2+^ influx *via* a protein kinase A-dependent pathway (Ito et al., 2019). NCX localization in dyads remains a controversial topic (Scriven et al., 2000; Thomas et al., 2003), but it is reported that at least a fraction of NCX is colocalized with RyRs (Mohler et al., 2005; Jayasinghe et al., 2009; Schulson et al., 2011; Wang et al., 2014; Figure 1). While the main function of NCX is extrusion of Ca^2+^ during relaxation (Louch et al., 2012), numerous reports have suggested that Ca^2+^ entry *via* NCX can also act in “reverse mode” to trigger SR Ca^2+^ release (Sipido et al., 1997; Henderson et al., 2004; Lines et al., 2006; Larbig et al., 2010). This mechanism is proposed to follow Na^+^ influx during the rising phase of the action potential, which creates driving force for NCX-mediated Ca^2+^ entry.

Besides ion channels, such as the LTCC and NCX, the t-tubular membrane is also enriched in molecules which critically regulate their structure and function. Notable examples are amphiphysin-2 (BIN1), junctophilin-2 (JPH2), and caveolin-3 (Cav-3) (Figure 1), which are involved in functions spanning t-tubule growth, t-tubule microdomain formation, and regulation of LTCC and RyR localization and activity. A detailed discussion of these important t-tubule regulators will be made in the following sections.

## T-Tubule Remodeling

### T-Tubule Remodeling Throughout Life

**FIGURE 2:**
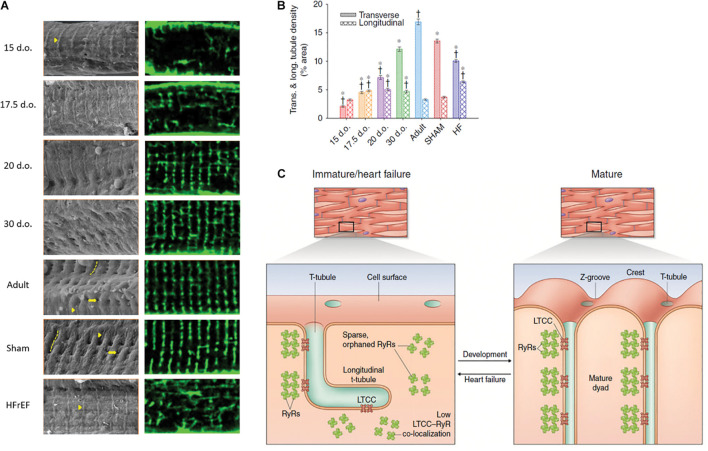
Changes in cardiomyocyte surface topography and dyadic organization in the developing and HFrEF rat heart. **(A)** Left panels: Scanning electron micrographs show the appearance of Z-spines (arrowheads) from an early time point during development and their re-appearance during post-infarction HFrEF. Z-grooves (dashed lines) and t-tubule openings (arrows) appear gradually during development and are lost in HFrEF. Right panels: temporally matched confocal captures of internal t-tubules (di-8-ANEPPS staining). **(B)** Quantification of these t-tubule signals illustrates an organizational shift from a predominantly longitudinal to largely transverse orientation during maturation, but reversion to an immature phenotype during HFrEF. **(C)** Structural parallels between developing and failing myocytes include not only t-tubule structure, but also similarly sparse arrangements of RyRs that exhibit low co-localization with LTCCs. d.o., days old. *P < 0.05 vs. adult in same category, ^†^*P* < 0.05 vs. SHAM in same category. Reproduced from Lipsett et al. (2019) with permission.

The process of t-tubule development varies between species (Ziman et al., 2010; Louch et al., 2015; Jones et al., 2018). In species such as cow, guinea pig, sheep and human, t-tubules have been found in the fetal stages of development (Forbes and Sperelakis, 1976; Sheldon et al., 1976; Forsgren and Thornell, 1981; Brook et al., 1983; Kim et al., 1992). A recent study in sheep specified that t-tubule development started at a late fetal stage (108 days out of 145 days), with further maturation occurring after birth (Munro and Soeller, 2016). In contrast, t-tubules only appear postnatally in mouse, rat, rabbit, and cat cardiomyocytes (Hirakow et al., 1980; Hoerter et al., 1981; Gotoh, 1983; Ziman et al., 2010; Hamaguchi et al., 2013; Lipsett et al., 2019). In rats, t-tubules appear gradually and become visible around 4–9 days after birth (Ziman et al., 2010; Mackova et al., 2017; Lipsett et al., 2019), with an initial appearance at the cell surface as sparse openings along Z-spines, with a rudimentary internal structure that is largely longitudinal in orientation (Figure 2). During further development, t-tubule density strikingly increases, with a re-orientation into a clear striated pattern at roughly 20 days of age, and a progressively more dominant presence of transverse over longitudinal elements (Ziman et al., 2010; Louch et al., 2015; Mackova et al., 2017; Figure 2). This process continues until surprisingly late stages of postnatal development (Oyehaug et al., 2013).

It is noteworthy that the heart exhibits robust contractile function at early embryonic stages of development in the absence of t-tubules (Brand, 2003). Since these embryonic myocytes are quite thin, EC coupling is sufficiently supported by U-shaped propagation of the Ca^2+^ transient from the surface of the cells to the interior (Rapila et al., 2008; Korhonen et al., 2010), reminiscent of Ca^2+^ release patterns observed in detubulated myocytes described above. This pattern of Ca^2+^ release is initiated at the cell surface, where LTCCs and RyRs are co-localized before birth (Snopko et al., 2008), and propagated by RyRs assembled on SR extensions positioned at intervals of 2 μm (de Diego et al., 2008; Rapila et al., 2008). These internal RyR clusters continue to be assembled along Z-lines as t-tubules arrive, and dyadic pairings with LTCCs are quickly formed (Ziman et al., 2010; Lipsett et al., 2019). Thus, dyadic functionality is rapidly attained once these structures are formed, allowing progressive increases in Ca^2+^ release efficiency with further maturation (Ziman et al., 2010; Lipsett et al., 2019).

Recent data suggest that the aging heart may exhibit reversal of the processes of dyadic assembly that take place during development. Aging is associated with reduced cardiomyocyte contractility, especially in males (Grandy and Howlett, 2006; Feridooni et al., 2015), and these changes are linked to reduction in cardiomyocyte t-tubule density (Kong et al., 2018a; Lyu et al., 2021). An accompanying reduction of t-tubule Ca^2+^ current density during aging has also been linked to loss of a cav-3-dependent mechanism that augments t-tubular Ca^2+^ current density (Kong et al., 2018a).

### T-Tubule Remodeling During Heart Failure

Heart failure (HF) is a leading cause of death worldwide. It is therefore of great importance to understand the different mechanisms underlying this condition, to facilitate discovery of novel treatment targets. HF can be divided into two main entities; HF with reduced ejection fraction (HFrEF) and HF with preserved ejection fraction (HFpEF). HFrEF describes a state where the cardiac muscle is unable to contract adequately, leaving the heart unable to meet the body’s oxygen demand. This state is associated with ventricular dilation, thinning of ventricular walls and high wall stress. HFpEF, on the contrary, is predominantly linked to cardiac hypertrophy, wall thickening, and maintained wall stress (Pieske et al., 2019). Although, ejection fraction is preserved in this condition, compromised cardiac chamber relaxation and filling yields impaired cardiac output. Hence, both HF entities are severe diseases with 2-year mortality rates between 14 and 19% (Lam et al., 2018).

**FIGURE 3:**
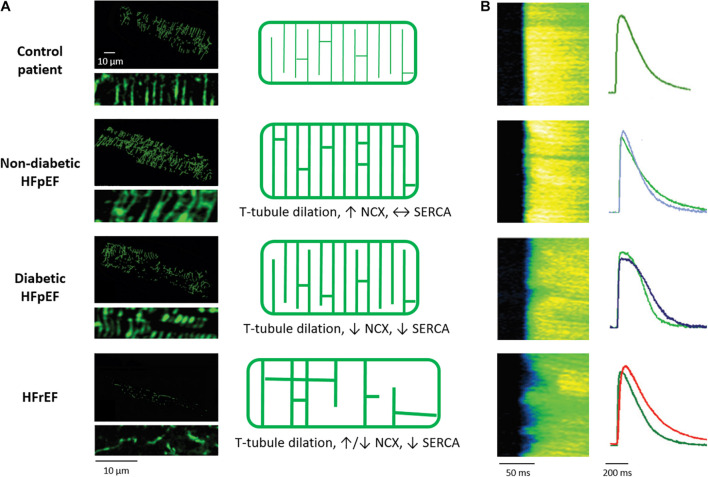
Alterations in cardiomyocyte t-tubule structure and Ca^2+^ release in different HF entities. **(A)** Left panels: 3D reconstructions and 2D zoom-ins of t-tubules imaged in human cardiac biopsies (wheat germ agglutinin staining). Healthy individuals are compared with HFpEF patients, with and without diabetes mellitus, and HFrEF patients. As illustrated in the schematics (right panels), t-tubule density is unchanged in diabetic HFpEF, increased in non-diabetic HFpEF, and decreased in HFrEF. In all HF entities, t-tubules dilate. However, whereas t-tubule remodeling is accompanied by increased NCX and SERCA function in non-diabetic HFpEF, Ca^2+^ removal by these proteins is decreased in diabetic HFpEF and HFrEF. **(B)** T-tubule disruption promotes dyssynchronous and slowed Ca^2+^ release in rats with HFrEF but not HFpEF, while slowed Ca^2+^ removal promotes diastolic dysfunction in diabetic HFpEF and HFrEF. For each disease state, representative Ca^2+^ transients (right panels) are presented with comparison to a control cardiomyocyte (green) for the individual rat models. Micrographs and Ca^2+^ transients are reproduced from Frisk et al. (2021) with permission.

HFrEF mechanisms have been extensively investigated, and numerous studies in a range of species and disease etiologies have linked disease progression to alterations in t-tubule structure and function (Jones et al., 2018; Figure 3). Typically this remodeling includes reduced t-tubule density (Maron et al., 1975; Heinzel et al., 2008; Swift et al., 2008; Wei et al., 2010; Wu et al., 2011, 2012; Xie et al., 2012; Ibrahim et al., 2013; Frisk et al., 2021; Yamakawa et al., 2021), including a lower density of transversely oriented tubules (Louch et al., 2006, 2013; Song et al., 2006; Swift et al., 2008; Ibrahim et al., 2010, 2013; Wei et al., 2010; van Oort et al., 2011; Wu et al., 2011; Lyon et al., 2012; Wagner et al., 2012; Xie et al., 2012; Oyehaug et al., 2013; Frisk et al., 2021). Based on this finding, it has often been claimed that t-tubules are “lost” in HFrEF. However, recent work has indicated that the t-tubule frame may in fact be maintained in this condition, although cell size increases (Frisk et al., 2021). Thus, the observed decrease in t-tubule density is suggested to reflect a lack of adaptive remodeling to meet the developing cellular hypertrophy, rather than a degradation of t-tubule structure *per se*. Further complicating the picture is the observation that t-tubule remodeling in HFrEF frequently includes an increased fraction of longitudinally oriented tubules (Kaprielian et al., 2000; Louch et al., 2006, 2013; Song et al., 2006; Sachse et al., 2012; Swift et al., 2012; Wagner et al., 2012; Oyehaug et al., 2013; Frisk et al., 2016, 2021), t-tubule dilation (Maron et al., 1975; Schaper et al., 1991; Kostin et al., 1998; Kaprielian et al., 2000; Lyon et al., 2012; Pinali et al., 2017), loss of tubule openings at the cell surface (Lyon et al., 2009; Lipsett et al., 2019), and the appearance of broad t-tubule “sheets” (Pinali et al., 2017; Seidel et al., 2017a; Fiegle et al., 2020). The animal models employed in these examinations have included myocardial infarction (Louch et al., 2006; Swift et al., 2008; Lyon et al., 2009; Wagner et al., 2012; Oyehaug et al., 2013; Crocini et al., 2014; Frisk et al., 2016, 2021; Sanchez-Alonso et al., 2016; Schobesberger et al., 2017; Lipsett et al., 2019), aortic stenosis (Wei et al., 2010; Ibrahim and Terracciano, 2013; Pinali et al., 2013; Caldwell et al., 2014), hypertension (Song et al., 2006; Xie et al., 2012; Shah et al., 2014; Singh et al., 2017), tachycardia (He et al., 2001; Balijepalli et al., 2003; Caldwell et al., 2014), and diabetes (Stolen et al., 2009; Ward and Crossman, 2014). Similar disruption of t-tubule structure has also been reported during chronic ischemia (Heinzel et al., 2008) and atrial fibrillation (Lenaerts et al., 2009). Notably, t-tubule remodeling is not limited to the left ventricle, as comparable alterations have been reported in the right ventricle (Wei et al., 2010; Xie et al., 2012) and atrial cells as well (Melnyk et al., 2002; Dibb et al., 2009; Lenaerts et al., 2009; Wakili et al., 2010; Richards et al., 2011). In disease models with a non-uniform myocardial affliction, such as myocardial infarction, there are reports that t-tubule remodeling exhibits a spatial gradient, with the most marked changes occurring proximal to the infarcted myocardium (Frisk et al., 2016; Pinali et al., 2017; Wang et al., 2018). Perhaps most importantly, examinations in human tissue (Crossman et al., 2015; Frisk et al., 2021) broadly concur with the findings described in animal disease models, as reviewed in Louch et al. (2010b), Ibrahim et al. (2011), Guo et al. (2013), and Hong and Shaw (2017).

T-tubule disorganization during HFrEF causes spatial dissociation between key players in EC coupling, most notably LTCCs and RyRs. This rearrangement leads to the formation of “orphaned” RyRs which are no longer co-localized with t-tubules (Louch et al., 2006; Song et al., 2006; Figure 2C). A resulting de-synchronization of the Ca^2+^ transient occurs, as Ca^2+^ release from orphaned RyRs can only be triggered after diffusion of Ca^2+^ from intact dyads (He et al., 2001; Balijepalli et al., 2003; Cannell et al., 2006; Kemi et al., 2011; Xie et al., 2012; Louch et al., 2013). Ca^2+^ release dyssynchrony is further augmented in failing cells by reduced SR Ca^2+^ content (Oyehaug et al., 2013). The net result of this de-synchronized Ca^2+^ transient is slowed and reduced magnitude of contraction; a hallmark of HFrEF (Louch et al., 2010b).

Impaired efficiency of CICR is not only linked to reorganized t-tubules and orphaned RyRs, but also a reduced ability of t-tubules to trigger Ca^2+^ release where they are present. This deficit is at least partly attributable to reduced t-tubular LTCC current (Bryant et al., 2015; Sanchez-Alonso et al., 2016; Lipsett et al., 2019). Reduced L-type current has, in turn, been linked to altered localization of LTCCs, but also changes in AP configuration, that prevent optimal channel recruitment (Sah et al., 2002; Louch et al., 2010a). Interesting data have suggested that there is also impairment of AP propagation into some t-tubules in failing cardiomyocytes, resulting in desynchronization of Ca^2+^ release (Crocini et al., 2014). Continuing work has suggested that this loss of electrical activation may be caused by constriction of t-tubule geometry in this condition (Scardigli et al., 2017; Uchida and Lopatin, 2018).

Recent work has indicated that loss of t-tubule functionality during HFrEF additionally stems from alterations on the SR side of the dyad. We observed “dispersion” of RyRs in MI-induced HFrEF, characterized by break-up of RyR clusters into smaller sub-clusters (Kolstad et al., 2018; Figure 2C). Functionally, we observed that this dispersion was associated with increased “silent” Ca^2+^ leak, not visible as sparks. Furthermore, we found that larger multi-cluster CRUs exhibited low fidelity Ca^2+^ spark generation. When successfully triggered, sparks in failing cells displayed slow kinetics as Ca^2+^ spread across dispersed CRUs. Previous work performed by us and others demonstrated that sparks occur almost exclusively at t-tubules (Brette et al., 2005; Meethal et al., 2007; Louch et al., 2013), suggesting that CRU dispersion and slow sparks solely occur at intact dyads. Thus, t-tubule and CRU remodeling may occur independently. Ultimately, it seems likely that t-tubule and CRU disruption have additive effects, yielding even more marked de-synchronization and slower SR Ca^2+^ release (Kolstad et al., 2018).

Importantly, not all dyadic changes during HFrEF are detrimental. For example, at early stages of disease, longitudinal tubules appear before transverse elements have disappeared (Louch et al., 2006). LTCCs are co-localized with RyRs at longitudinal dyads, allowing these structures to actively release Ca^2+^ (Lipsett et al., 2019). Thus, their early growth following the initiating insult (for example myocardial infarction or induction of aortic banding) is thought to be compensatory (Mork et al., 2007). At later time points, contractile function declines as transverse elements are lost, supporting that this latter type of remodeling is a direct cause of HFrEF (Wei et al., 2010; Shah et al., 2014).

### Heart Failure With Preserved Ejection Fraction

The above discussion has highlighted convincing evidence that disrupted t-tubule structure and Ca^2+^ homeostasis are a root cause of HFrEF. However, approximately 50% of HF patients exhibit HFpEF (Lekavich et al., 2015), and the mechanisms underlying this disease entity are merely beginning to be unraveled. Recent evidence from our group indicates that a distinct form of subcellular remodeling occurs in this condition. Using patient biopsies we observed that, conversely to HFrEF, cardiomyocytes from HFpEF individuals exhibited increased t-tubule density (Figure 3A), and a positive correlation between t-tubule density and the severity of *in vivo* diastolic dysfunction (Frisk et al., 2021). Higher t-tubule densities resulted from a combination of t-tubule dilation and proliferation, consistent with adaptive remodeling. These data contribute to growing evidence that t-tubules have compensatory capacity, as similar increases in t-tubule density have been reported during physiological hypertrophy following exercise training (Kemi et al., 2011). In this sense, t-tubule growth during concentric, non-dilatory hypertrophy may be viewed as a continuation of processes set in motion during development. Importantly, functional data support such a compensatory role, as cardiomyocytes obtained from hypertensive and ischemic rat models of HFpEF revealed maintained Ca^2+^ release and reuptake despite decreased SERCA protein levels (Figure 3B). Marked phosphorylation of the SERCA inhibitory protein phospholamban was identified as the key motif for unchanged diastolic Ca^2+^ homeostasis (Frisk et al., 2021). Other studies examining Dahl salt sensitive rats and aorta banded rats with impaired cardiac relaxation have reported similar maintenance of t-tubule structure and Ca^2+^ homeostasis (Roe et al., 2017; Curl et al., 2018; Kilfoil et al., 2020).

HFpEF includes a set of patients with diverse etiologies, including those suffering from cardiac ischemia or hypertension, as noted above, but also diabetes (Lekavich et al., 2015). In contrast to other disease etiologies, diabetic HFpEF appears to negatively affect both t-tubule integrity and Ca^2+^ handling. Indeed, we observed that HFpEF patients and animal models with diabetes exhibited less compensatory t-tubule growth than their non-diabetic counterparts, as t-tubule density was merely maintained (Frisk et al., 2021; Figure 3A). We hypothesize that this reduced capacity for adaptive remodeling may be related to abnormal caveolin-3 and/or phospoinositol-3 kinase expression and activity, since cholesterol, fatty acid and phosphoinositide composition of the sarcolemmal membrane are altered during diabetes (Russell et al., 2017). As discussed later, decreased autophagy might also play a role (Hsu et al., 2016; Seidel et al., 2019). Despite less adaptive t-tubule remodeling, systolic Ca^2+^ release was observed to be quite well-maintained in diabetic HFpEF. However, diastolic dysfunction in this condition was linked to impairment of diastolic Ca^2+^ homeostasis, caused by reduced activity of both SERCA and NCX (Frisk et al., 2021; Figure 3B). Accumulating evidence has indicated that the reduced NCX activity in diabetic HFpEF may be related to elevated cytosolic Na^+^ levels, which reduce the driving force for t-tubular Ca^2+^ removal. This Na^+^ accumulation has in turn been linked to increased activity of the Na^+^-glucose co-transporter 1 (Lambert et al., 2015; Frisk et al., 2021) and/or the Na^+^-H^+^-exchanger (Jaquenod De Giusti et al., 2019). Thus, there appears to be impairment of diastolic Ca^2+^ homeostasis in diabetic HFpEF, which includes detrimental alterations in t-tubule function.

While investigations of HFpEF mechanisms are ongoing, the above studies present an emerging view that t-tubule structure remains adequate to maintain near-normal SR Ca^2+^ release in this condition. This striking difference from the cardiomyocyte phenotype linked to HFrEF is perhaps not surprising. In addition to the aforementioned differences in chamber remodeling, HFrEF is primarily associated with activation of the renin-angiotensin- aldosterone system and stretch-mediated signaling pathways, while inflammation, endothelial dysfunction, and alteration of the extracellular matrix are key components of HFpEF (Paulus and Tschope, 2013). Perhaps most importantly, established therapies for HFrEF, such as neurohumoral blockage, have proven ineffective for treatment of HFpEF (Paulus and Tschope, 2013). Thus, there appears to be a growing consensus that management of HFpEF will require novel strategies, and that these therapies may be best directed at non-cardiomyocyte alterations occurring in the endothelium and/or extracellular matrix.

### Consequences for Arrhythmia

Alterations in t-tubule structure and function during HF not only impact systolic and diastolic function, but also have complex consequences for arrhythmia generation (Orchard et al., 2013). HFrEF is frequently associated with increased RyR “leak,” which can be exacerbated by SR Ca^2+^ overload during β-adrenergic stimulation (Dridi et al., 2020). Extrusion of this spontaneously released Ca^2+^ by NCX triggers an inward, depolarizing current. If this current occurs during the downstroke of the action potential, an early afterdepolarization (EAD) results, while delayed afterdepolarizations (DADs) are triggered by spontaneous Ca^2+^ release between beats (Orchard et al., 2013). How would t-tubule remodeling during HFrEF affect these processes? As noted above, t-tubule reorganization in this disease results in the formation of orphaned RyRs. However, Ca^2+^ sparks occur mostly at intact dyads where t-tubules are present, and only to a much lesser degree at orphaned RyRs (Brette et al., 2005; Meethal et al., 2007; Louch et al., 2013). In the event that spontaneous release does occur at an orphaned RyR, an EAD or DAD would be less likely to occur because NCX is more distally located (Biesmans et al., 2011; Edwards and Louch, 2017). On the other hand, propagation of spontaneously released Ca^2+^ as a wave is more likely to occur, since distally localized NCX does not draw Ca^2+^ away from the wavefront. Recent data have painted a more complex picture that considers also the relationship between Ca^2+^ release and LTCC activity. Less Ca^2+^-dependent inactivation of LTCCs, due to displaced (orphaned) RyRs, and resulting increased Ca^2+^ influx has been predicted to contribute to SR Ca^2+^ overload (Shiferaw et al., 2020). Thus, t-tubule remodeling may make spontaneous Ca^2+^ release more likely in HFrEF, while at the same time attenuating the link between these events and EADs/DADs.

T-tubule remodeling also has complex implications for EADs triggered by channel reopening. Phase-2 EADs initiated by LTCCs may be favored in HFrEF, as L-type current is redistributed to the cell surface (Sanchez-Alonso et al., 2016; Loucks et al., 2018). This increase in LTCC open probability has been linked to calcium-calmodulin kinase II-dependent phosphorylation of the channel, which augments window current (Sanchez-Alonso et al., 2016), but also phosphorylation by protein kinase A, based on the presence of β2 adrenergic receptors and phosphodiesterases (Loucks et al., 2018). Conversely, loss of LTCCs and NCX in t-tubules, and their respective currents, may shorten the AP making phase-2 EADs less likely, but Na^+^ channel re-activation and phase-3 EADs more likely (Orchard et al., 2013; Edwards and Louch, 2017). A shorter AP, and accompanying reduction in refractory period, also increases the chance of re-entrant arrhythmia (Herring et al., 2019). In addition to changes in ion channel expression and regulation, Hong and colleagues have provided evidence that alterations in the geometry of the t-tubules themselves can promote the occurrence of EADs (Hong et al., 2014). They observed that intricate folding of the t-tubule lumen creates a microenvironment with slowed ion diffusion. Upon loss of these membrane folds, more rapid exchange of ions between the t-tubule lumen and extracellular space was linked to prolonged action potential duration, and the generation of EADs and arrhythmia (Hong et al., 2014). This interesting observation raises the possibility that t-tubule dilation observed during HFrEF and HFpEF (Figure 3A) may also have arrhythmogenic consequences.

Finally, growing evidence supports that t-tubule remodeling during HFrEF can promote pro-arrhythmic alternans. Two mathematical modeling studies have linked spatially discordant alternans to the increased fraction of orphaned RyRs that yield a phase of secondary release following Ca^2+^ diffusion from intact dyads (Li et al., 2012; Song et al., 2018). Jiang et al. (2014) suggested that there is, however, an intermediate range of t-tubule remodeling where this phenomenon occurs. Thus, failing ventricular myocytes and healthy atrial myocytes are likely susceptible to this mechanism of alternans, while failing atrial myocytes may be less prone due to their very low t-tubule densities (Jiang et al., 2014). Importantly, since Ca^2+^-voltage coupling is an additional determinant of alternans generation (Gaeta et al., 2009), the susceptibility of failing myocytes to alternans is expected to be complicated by changes in AP configuration. Interrogating and integrating these mechanisms will be an important topic of future work.

## Emerging T-Tubule Regulators

The above sections have highlighted a growing consensus that t-tubule remodeling is a key trigger for reduced contractility and arrhythmia in HFrEF. This understanding has led to a concerted effort to reveal the processes that control t-tubule structure in both health and disease. We review the emerging findings from this work in the following sections.

### Workload

Accumulating evidence has indicated that ventricular workload plays a key role in regulating t-tubule structure. Pioneering work by the Terraciano group was the first to indicate that the loss of t-tubules during HFrEF could be directly triggered by the elevated ventricular workload (reviewed in Ibrahim and Terracciano, 2013). In their work, failing hearts were unloaded by heterotopic transplantation into healthy animals, resulting in rescue of t-tubular structure (Ibrahim et al., 2012). Similar approaches to unloading of the failing heart either pharmacologically (Chen et al., 2012; Xie et al., 2012; Huang et al., 2016) or *via* resynchronization therapy (Sachse et al., 2012; Lichter et al., 2014) have similarly shown improved cardiac function linked to restoration of t-tubules. Interestingly, unloading of healthy hearts promoted loss of t-tubules (Ibrahim et al., 2012). These findings support the notion that there is an optimal range of loads necessary to maintain t-tubule integrity (Ibrahim et al., 2015).

Workload is a broad term, and ongoing efforts are aimed at revealing the precise critical mechanical stimuli which control t-tubule structure. The dilated, thin-walled ventricle leads to elevated ventricular wall stress in HFrEF, and this appears to be a critical mechanical signal. Indeed, when we examined regional differences across the post-infarction rat heart we observed a correlation between wall stress and t-tubule disruption. This remodeling included significant t-tubule disruption near the infarct, where thinning of the myocardium markedly increases wall stress, together with locally impaired Ca^2+^ homeostasis and *in vivo* systolic and diastolic dysfunction (Frisk et al., 2016; Roe et al., 2019). *Ex vivo* studies supported this direct role of wall stress in the regulation of t-tubular structure. A likely implication of this wall stress-t-tubule relationship is in differentiating the pathophysiology of HFrEF and HFpEF, since as noted above, HFpEF is associated with concentric remodeling and maintained wall stress, and maintained t-tubule density (Frisk et al., 2021).

How does mechanical overload lead to the t-tubule loss? What are the associated mechanosensors and signaling pathways? While the precise mechanisms continue to be investigated, titin may be of importance, as it plays a key role in cardiomyocyte mechanotransduction by regulating interactions between the extracellular matrix and sarcomeres (Linke, 2008). Telethonin, or titin cap (TCap), a stretch-sensitive protein located in the Z-disc of cardiomyocytes, also integrates mechanical signals (Ibrahim and Terracciano, 2013; Ibrahim et al., 2013). Indeed, TCap knockout mice exhibited progressive disruption of the t-tubules during development (Ibrahim et al., 2013), and TCap downregulation is reported in HFrEF (Lyon et al., 2012). Furthermore, increased Tcap expression was associated with recovery of t-tubules during reverse remodeling induced by SERCA2a gene therapy (Lyon et al., 2012). While further studies are needed to examine the interplay between myocardial load, expression of Tcap, and t-tubule organization, this protein is viewed as a promising therapeutic target (Roe et al., 2015).

Other studies have linked membrane-mediated mechanosensation by stretch-activated channels, integrins, proteoglycans and angiotensin II type I receptors to activation of a variety of pathways, including MAPK, AKT, calcineurin-NFAT, and microRNA-24 (miR-24) (Lammerding et al., 2004; Dostal et al., 2014). Of these, it should be noted that miR-24 is a member of the miR23a-27a-24-2 cluster, which is upregulated in HF (Xu et al., 2012). Xu et al. (2012) observed that overexpression of miR-24 disrupted dyadic structure, and reduced CICR efficiency. This group also found that miR-24 suppression protected against HFrEF progression (Li et al., 2013).

As Jones et al. (2018) discussed in their recent review article, elevated workload and wall stress regulate not only cardiomyocyte remodeling, but also the extracellular matrix. Interestingly, recent evidence indicates that fibrosis occurs within t-tubules in HFrEF, but not in HFpEF where wall stress is maintained (Crossman et al., 2017; Frisk et al., 2021). Crossman and colleagues in fact detected fibroblast filopodia within the t-tubules of HFrEF hearts, suggesting a mechanism for local collagen production (Crossman et al., 2017). Although the consequences of this collagen deposition are unclear, it is proposed that accompanying stiffening of the membrane might mark the t-tubule for degradation (Louch and Nattel, 2017). In apparent support of this hypothesis, it should be noted that in the post-infarcted heart, the most marked t-tubule loss occurs in regions proximal to the infarct, where the fibrosis is most pronounced (Frisk et al., 2016; Seidel et al., 2017b). Fibrosis is expected to impair mechanosignaling, and new data indicate that this might occur within t-tubules themselves during stretch and contraction (Dyachenko et al., 2009; McNary et al., 2011). Such a role is suggestive of t-tubules having a self-regulating feature.

In summary of the above discussion, it appears to be no coincidence that the vast majority of present HFrEF therapies relieve symptoms and delay disease progression by reducing the workload of the heart, and specifically the physical stress placed on the ventricular wall (Cohn, 1996; Tarzia et al., 2016; Berliner and Bauersachs, 2017; Schmitto et al., 2018; Vaidya and Dhamoon, 2019). However, there is great potential for improvement. For example, wall stress and t-tubule structure might be longitudinally tracked as biomarkers, aimed at optimizing ventricular load. Future treatment strategies could also be envisioned which directly inhibit the mechanosensing that signals t-tubule remodeling.

### Insight From Developing Cardiomyocytes

In the quest to unravel signaling pathways involved in triggering subcellular remodeling in HFrEF, newfound attention has turned to the importance of the fetal gene program. Indeed, our group has recently observed striking similarities between developing and diseased cardiomyocytes (Figure 2). Structurally, these similarities include a disorganized and predominantly longitudinal t-tubule configuration in both types of cells. There also appear to be similarities in dyadic configuration, as these junctions are progressively “packed” with LTCCs and RyRs in developing cardiomyocytes and “unpacked” in HFrEF, consistent with a “last in, first out” paradigm (Lipsett et al., 2019). However, even though immature and failing cardiomyocytes share rather similar subcellular structure, functional differences were observed. For example, while dyads were observed to effectively trigger Ca^2+^ release from early developmental states, impaired release Ca^2+^ release was noted along t-tubules in failing cells (Lipsett et al., 2019). Thus, it is unlikely that there is a complete reversion of subcellular structure/function to an immature state in this disease.

**FIGURE 4:**
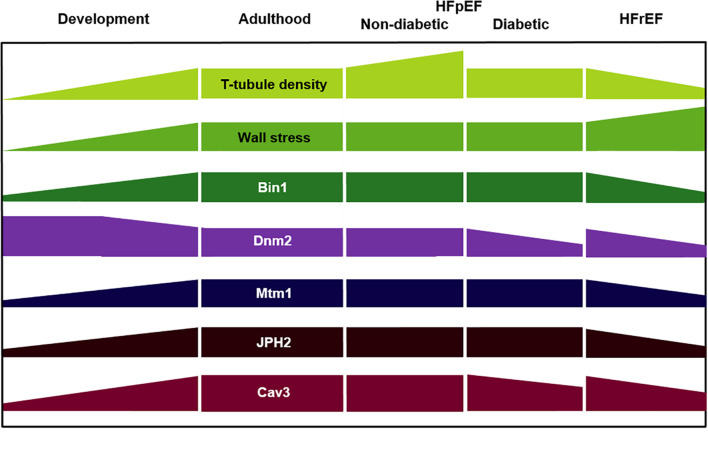
Key regulators of t-tubule structure during development, adulthood, and heart failure of various etiologies. HFpEF, heart failure with preserved ejection fraction; HFrEF, heart failure with reduced ejection fraction; Dnm2, dynamin-2; Mtm1, myotubularin-1; JPH2, junctophilin-2; Cav3, caveolin-3.

The above observations suggest that subcellular remodeling during HFrEF progression likely shares signaling mechanisms with the developing heart, which may include fetal genes that are reactivated in disease, and/or adult genes that are suppressed (Rajabi et al., 2007; Louch et al., 2015; Figure 4). Until recently, however, these signals and cardiomyocyte developmental biology in general have been rather under-investigated. This has changed as interest in the generation of stem cell-derived cardiomyocytes and myocardium has come to the forefront, and included an ever-expanding use of human induced pluripotent stem cells (iPSCs). Most early work on iPSC-derived cardiomyocytes generated cells with quite immature features, including a round instead of rod shape, lack of t tubules, poor cooperation of LTCCs and RyRs, and dyssynchronous Ca^2+^ transients with delayed Ca^2+^ release occurring in the cell center. More recently, however, improved differentiation has been achieved by treating iPSCs with hormones thought critical for cardiac maturation. Parikh et al. (2017) observed that supplementing the culture medium with thyroid and glucocorticoid hormones, followed by single-cell culture on a Matrigel mattress, stimulated rudimentary t-tubule development and functional coupling of LTCCs and RyRs. Similarly, Huang et al. (2020) documented t-tubule formation close to z-lines in iPSC-derived cardiomyocytes treated with thyroid hormone, dexamethasone, and insulin-like growth factor-1 in both 2D and 3D culture conditions. It is noteworthy that both of these studies included a cellular environment which provided mechanical cues, reinforcing the view expressed above that t-tubule growth and maintenance are highly workload-sensitive. Indeed, even without hormone treatment, Silbernagel et al. (2020) found that reshaping single iPSC-CMs in rectangular 3D-micro-scaffolds triggered t tubule formation and improved Ca^2+^ handling. Ronaldson-Bouchard et al. (2018) on the other hand, co-cultured hiPSC-CMs with human cardiac fibroblasts, exposed the developing tissue to mechanical load, and gradually increased electrical stimulation. With this method, the authors observed an impressive degree of cellular maturation, including transversely oriented t-tubules, functional Ca^2+^ handling, and adult-like gene expression profiles. This progress has given credence to the application of iPSC-derived cardiomyocytes for human cardiac disease modeling, drug development, and eventually, engineered cardiac tissue which may be suitable for *in vivo* implantation.

### BIN1 and Its Partners

In addition to the broader t-tubule regulatory processes described above, several specific proteins have been attributed roles in dyadic structure and function. Of these, BIN1 has received particular attention. This membrane sensing and bending protein has been identified as a key regulator of t-tubule structure assembly and maintenance in both developing and diseased hearts (Lee et al., 2002; Muller et al., 2003; Hong et al., 2010, 2014; see Figure 4). However, understanding of these roles has been complicated by the fact that BIN1 is expressed in several tissue- and species-specific isoforms. Early work suggested that the presence of exon 11 is required to induce tubulogenesis (Lee et al., 2002; Kojima et al., 2004), and exon 11-containing BIN1 isoforms expressed in rat, sheep, and human myocardium have indeed been observed to induce t-tubule formation (Caldwell et al., 2014; De La Mata et al., 2019; Lawless et al., 2019; Li L. L. et al., 2020). However, other studies have reported that exon 11 is dispensable for t-tubule development (Prokic et al., 2020), and shown that the mouse heart expresses four BIN1 splice variants which do not contain this motif (BIN1, BIN1+13, BIN1+17, and BIN1+13+17) (Forbes and Sperelakis, 1976; Hong et al., 2014). In addition to gross t-tubule biogenesis, there may also be isoform-specific roles in determining their fine structure and function. Hong and colleagues reported that isoform BIN1+13+17 creates microdomains by folding the tubular inner membrane (Hong et al., 2014), and attracts phosphorylated RyRs on the SR membrane (Fu et al., 2016). Since BIN1 also anchors microtubules transporting LTCCs, in a process known as targeted delivery (Hong et al., 2012b; De La Mata et al., 2019), an emerging view is that this protein serves as a master regulator of dyadic structure and function. Consistent with this paradigm, genetic knockout of BIN1 has been shown to be embryonically lethal (Muller et al., 2003).

Accumulating data have identified BIN1 as a culprit in cardiac pathology, with its downregulation linked to decreased t-tubule density in HFrEF (Caldwell et al., 2014; Hong et al., 2014; Figure 4). An associated reduction in t-tubule folding has also been predicted to augment diffusion of ions within individual t-tubules which, as noted above, may predispose for cardiac arrhythmias (Hong et al., 2014). Furthermore, given BIN1’s proposed role in trafficking of LTCCs and RyRs to the dyad, it seems highly plausible that “unpacking” of these proteins during HFrEF progression may be linked to declining BIN1 levels (Manfra et al., 2017). These findings indicate that BIN1 may serve as a therapeutic target in HFrEF, and preclinical data support this view. Treating mice with isoproterenol-induced HFrEF with adenoviral BIN1 overexpression was shown to attenuate hypertrophy, increase t-tubular microfolding, normalize SERCA2a distribution, and decrease the LTCC-RyR nearest neighbor distance (Liu Y. et al., 2020). This group additionally showed that BIN1 transduction rescued pre-existing global cardiac global dysfunction following aortic banding (Li J. et al., 2020). Beyond BIN1 overexpression as a therapeutic alternative, it appears that this protein may even serve as a biomarker. With normal, continuous turnover of BIN1 from dyads in healthy human patients, high levels of BIN1 are maintained in the blood. Thus, lowering of BIN1 levels has been linked to HF and arrhythmia (Hong et al., 2012a), including HFpEF disease severity and hospitalization risk (Nikolova et al., 2018), and HFrEF-associated risk of cardiovascular events (Hitzeman et al., 2020).

Given the exciting basic science and clinical data described above, more thorough investigation of BIN1’s protein partners seems warranted. Evidence from skeletal muscle has indicated that BIN1’s interaction with dynamin-2 (Dnm2) critically regulates tubulogenesis (Lee et al., 2002; Picas et al., 2014). This membrane-bound GTPase mediates membrane fission of clathrin-coated pits and plays a central role in membrane and vesicle trafficking (Gonzalez-Jamett et al., 2013). Dnm2 knockdown was found to rescue perinatal death in Bin1 knockout mice, and to normalize t-tubule formation and muscle function in animals with myopathies caused by Bin1 mutations (Tasfaout et al., 2017). An inhibitory role of Dnm2 in tubulogenesis was further supported by the observation that increasing its expression disrupts Bin1-induced tubulation in skeletal muscle (Gibbs et al., 2014).

**FIGURE 5:**
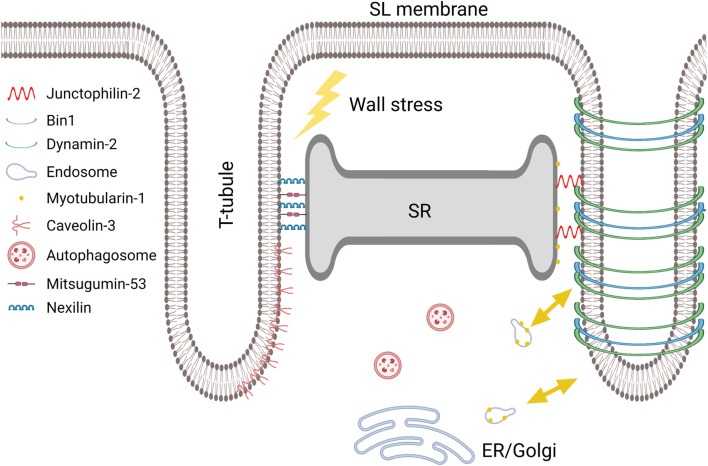
Proposed proteins and pathways involved in dyadic biogenesis, maintenance, and disruption. Created with BioRender.com.

A key aspect of Bin1’s function appears to be its ability to cluster phosphoinositides (PIs), in particular PI4, 5P_2_ (Lee et al., 2002). The dominant precursors of this phosphoinositide are PI and PI5P, which are created through the actions of the PI3P phosphatase myotubularin-1 (MTM1) (Ketel et al., 2016). This protein is expressed in most tissues, where it regulates endolysosomal sorting and trafficking by controlling PI expression patterns (Ketel et al., 2016; Figure 5, yellow arrows). Exciting data from the skeletal muscle field indicate that Bin1-mediated tubulogenesis is dependent on MTM1’s phosphatase activity, as MTM1 expression levels predict the extent of t-tubule biogenesis (Al-Qusairi and Laporte, 2011; Royer et al., 2013). Although MTM1’s role in t-tubule formation in cardiomyocytes is not yet resolved, PIs are indeed thought to be important for maintaining t-tubule integrity (Wu et al., 2011) and MTM1 dysregulation can induce dilated cardiomyopathy (Agrawal et al., 2014). Collectively, an emerging view is that balanced expression and activity of Bin1, Dnm2 and MTM1 are crucial for controlling tubule growth and maintenance (Figures 4, 5).

### Junctophilin-2

As noted in the first chapter, JPH2 is a membrane anchoring protein, important for connecting the sarcolemma and its t-tubules to the SR in the dyad (Takeshima et al., 2000; Minamisawa et al., 2004; Han et al., 2013; Beavers et al., 2014; Figure 5). In this capacity, JPH2 maintains dyadic dimensions, and efficient crosstalk between LTCCs and RyRs. Importantly, JPH2 also interacts with both LTCCs and RyRs (Jiang et al., 2016; Reynolds et al., 2016), although the precise nature of these connections is not completely understood.

As with BIN1, JPH2 has been shown to play a key role in the development of t-tubules and dyads, both pre- and postnatally (Ziman et al., 2010; Munro and Soeller, 2016; Figure 4). This function is enabled by the membrane on receptor nexus (MORN) motif of JPH2, which allows anchoring to the t-tubule membrane, while the carboxy terminal is secured within the lumen of the SR (Nishi et al., 2000; Takeshima et al., 2000). The role of JPH2 in forming dyads is supported by the parallel appearance of JPH2 and t-tubules along z-lines in advance of developing t-tubules (Ziman et al., 2010). Furthermore, in mouse studies, reduced expression of JPH2 has been found to prevent t-tubule growth or result in an immature longitudinal configuration (Chen et al., 2013; Reynolds et al., 2013), consistent with a role of JPH2 in anchoring transverse, but not longitudinal elements (Chen et al., 2013). When JPH2 is fully knocked out in mice, it results in embryonic mortality, suggesting that it is required for dyad formation at the surface of the cell, even before the development of t-tubules starts (Takeshima et al., 2000; Franzini-Armstrong et al., 2005; Jones et al., 2018).

In adult failing cardiomyocytes, declining expression of JPH2 has been linked to t-tubule remodeling and disrupted dyads (Minamisawa et al., 2004; Xu et al., 2007; Wei et al., 2010; Landstrom et al., 2011; Chen et al., 2012; Xie et al., 2012; Zhang et al., 2014; Frisk et al., 2016; Figure 4). A causative role of JPH2 as a promoter of this remodeling is supported by studies showing that overexpression of JPH2 protects against t-tubule degradation, abnormal SR Ca^2+^ release, and HFrEF (Guo et al., 2014; Reynolds et al., 2016).

Emerging data support the role of JPH2 as a regulator of dyadic proteins. JPH2 overexpression was reported to result in the formation of larger RyR clusters within CRUs (Munro et al., 2016), while Wang et al. (2014) observed a reduction in RyR and NCX co-localization following JPH2 knockdown. JPH2 binding to RyRs may directly stabilize the channel’s function, as JPH2 knockdown induced RyR hyperactivity (van Oort et al., 2011), while overexpression inhibited Ca^2+^ sparks (Munro et al., 2016). JPH2 is also reported to modulate LTCC activity, which has important implications for L-type current in HFrEF, where JPH2 expression is lowered (Jiang et al., 2016). Interestingly, recent data from Gross et al. (2021) indicate that the “Joining Region” of JPH2 may exert direct effects on the localization of LTCC in t-tubules, indicating that not only the presence of JPH2 but specifically JPH2-LTCC binding is necessary for maintenance of dyadic integrity and Ca^2+^ homeostasis.

What causes JPH2 changes during disease? A suggested mechanism for suppression of JPH2 expression is *via* upregulation of microRNA-24 (miR-24), as Xu et al. (2012) showed that a miR-24 antagomir protected against JPH2 downregulation and associated changes in t-tubule architecture. Mislocalization of JPH2 in the failing heart has also been reported, and linked to reorganization of the microtubules necessary for its delivery to dyads (Zhang et al., 2014; Prins et al., 2016).

Calpain cleavage of JPH2 is believed to be one of the mechanisms behind its downregulation, as first reported in mouse models of reversible heart failure (Guo et al., 2015), and ischemia-reperfusion injury (Wu et al., 2014). Furthermore, Wang et al. (2018) demonstrated increased calpain-mediated JPH2 cleavage in several mouse HFrEF models (myocardial infarction, transaortic banding, and chronic isoproterenol infusion), and found that inhibition of calpain partially restored both JPH2 expression levels and t-tubular density. These authors also observed that in a dual overexpression model of calpain and JPH2, JPH2 expression was only transiently maintained, and that subsequent deterioration in t-tubules and higher risk of cardiac death temporally correlated with declining JPH2 levels. Interestingly, contrasting reports also indicate that two different cleavage products of JPH2 may relocate to the nucleus, and attenuate (Guo et al., 2018b), or exacerbate cardiomyocyte stress responses (Lahiri et al., 2020).

Finally, the function of JPH2 has been shown to be dependent on its phosphorylation status (Guo et al., 2015), which is in turn regulated by “striated muscle preferentially expressed protein kinase” (SPEG). SPEG is downregulated in HFrEF, and loss of SPEG-dependent phosphorylation of JPH2 is suggested to promote t-tubule disruption (Quick et al., 2017).

Taken together, there is an abundance of evidence to support that JPH2 is a critical regulator of dyadic assembly and maintenance, and that this function extends far beyond a passive role in anchoring membrane positions. Thus, JPH2 is a promising target for future therapies.

### Caveolin-3

Cav-3 is known to play a key role in the formation of t-tubules and caveolae (Parton et al., 1997; Figures 4, 5). However, as with other dyadic regulators, emerging data suggest that decreased expression of Cav-3 has important functional consequences in the failing heart. Data from the Orchard group have shown that Cav-3 knockout mice exhibit cardiac dysfunction, associated with t-tubule reorganization and decreased LTCC current density (Bryant et al., 2018b). In another study, this group found that pressure overload in mice triggered loss of t-tubular Ca^2+^ current density and impairment of Ca^2+^ release, linked to lowered levels at Cav-3 (Bryant et al., 2018a). Interestingly, follow-up work with Cav-3 overexpressing mice exposed to pressure overload showed cardioprotective effects linked to maintained t-tubular Ca^2+^ current (Kong et al., 2019). Of note, these protective effects may also be linked to associations between Cav-3 and JPH2, as Cav-3 overexpression reportedly stabilizes JPH2, and thus t-tubules (Minamisawa et al., 2004).

### Autophagy

Recent work has identified a role of autophagy in regulating the assembly and disassembly of cardiac t-tubules (Figure 5). First, dexamethasone, a synthetic glucocorticoid, was shown to aid t-tubule growth in stem cell-derived cardiomyocytes (Parikh et al., 2017), and knockout of cardiac glucocorticoid receptors was found to induce heart failure and disturbances in Ca^2+^ handling (Oakley et al., 2013). However, it wasn’t until Seidel and colleagues (Seidel et al., 2019) recently examined the t-tubule network in glucocorticoid receptor knockout mice that the former results were linked to autophagy. Here they demonstrated that t-tubule loss caused by glucocorticoid receptor knockout can be rescued by treatment with dexamethasone, and that this is associated with upregulation of the autophagy markers LC3BII and p62. Furthermore, treatment with rapamycin, an autophagy enhancer, reproduced the findings with dexamethasone treatment. Conversely, treatment with chloroquine and bafilomycin A1 (autophagy blockers) exacerbated detrimental effects on t-tubules (Seidel et al., 2019). In line with these findings, it has also been shown that a high-fat diet, and diabetes, induce apoptosis and cardiac alterations through inhibition of autophagy (Hsu et al., 2016).

### Nexilin

Very recently, several studies from the Chen group have identified an exciting new dyadic regulator, called Nexilin (NEXN) (Figure 5). Previously known as an actin-binding and Z-disk protein, NEXN was shown to be critical to the formation of dyadic membranes, as myocytes from knockout animals did not develop t-tubules and exhibited early postnatal cardiomyopathy and lethality (Liu et al., 2019). Furthermore, conditional knockout of NEXN in adult cardiomyocytes resulted in a remodeling of t-tubule structure that was highly reminiscent of HFrEF, with loss of transverse t-tubules and an increased proportion of longitudinal elements (Spinozzi et al., 2020). The authors additionally reported that NEXN interacts with both JPH2 and RyRs, and that its loss results in decreased expression of these and other dyadic proteins, and accompanying impairment of Ca^2+^ homeostasis (Liu et al., 2019; Spinozzi et al., 2020). These findings are of considerable interest, as nexilin mutations are linked to cardiopathy in mice and humans (Hassel et al., 2009; Wang H. et al., 2010; Haas et al., 2015; Liu C. et al., 2020). Further work is required to determine the exact mechanism by which NEXN grows and maintains dyads, and whether NEXN alterations occur in acquired HFrEF. In this regard, it is noteworthy that NEXN expression levels were found to be maintained in HFrEF patients (Chen et al., 2018).

### Mitsugumin 53

The muscle-specific membrane repair protein mitsugumin 53 (MG53) is an up and coming t-tubule regulator (Kitmitto et al., 2019; Figure 5). This “wound-healing” protein is part of the tripartite motif family (TRIM), and is often referred to as TRIM72. Wang X. et al. (2010) were the first to identify MG53 as critical for maintaining cardiomyocyte sarcolemmal stability. The sarcolemmal membrane of the cardiomyocyte is the first line of defense against external stresses, such as oxygen and nutrient deprivation, inflammation and oxidative stress, and indeed, loss of sarcolemmal viability is a key step in cell death *via* necrosis (Kitmitto et al., 2019). Regulatory injury-repair proteins, such as MG53, are thus important for the integrity of both the sarcolemma and the cell as a whole. A new study suggests that while MG53 is not necessary for the development or maintenance of t-tubules during health, it may crucially preserve their integrity and function when the heart is placed under pathological stress (Zhang et al., 2017). When an injury or defect appears in the cell membrane, MG53 travels to the injury site and “plugs the hole,” by re-sealing the membrane (Cooper and McNeil, 2015). MG53 coordinates this role through interplay with caveolaer proteins (Kitmitto et al., 2019). He et al. (2012) found that elevated expression of MG53 improved heart function and augmented membrane repair capacity. While these authors did not specifically link these findings to changes in t-tubule structure/function, such effects seem likely based on the well-established role of declining t-tubule integrity in disease.

### Protein Kinase C

Protein kinase C (PKC) activation has been implicated in t-tubule remodeling in a recent study by Guo et al. (2018a), where the authors found a transient elevation in PKC activity in the days following transaortic banding. Inhibition of PKC during this period ameliorated subsequent t-tubule remodeling and heart failure development. It was shown that the increase in PKC coincided with a transient biphasic mode of actin depolymerization and repolarization, and that PKC inhibition abolished this response. Furthermore, the use of various inhibitors or stabilizers of F-actin polymers seemed to protect the t-tubule system after aortic banding, indicating that the sudden biphasic response is detrimental to t-tubule integrity. Blocking stretch-activated channels diminished the PKC-mediated effect on t-tubules, implicating these channels in mechanosensitive regulation of t-tubule structure downstream of PKC. These exciting findings are consistent with a growing appreciation for the importance of workload in regulating t-tubule structure, but are the first to implicate a role of PKC and actin filament dynamics in these processes.

### Integrated Understanding of T-Tubule Regulators

The above discussion has highlighted recent work implicating a plethora of proteins involved in the growth and maintenance of t-tubules and their remodeling during disease. How should we make sense of this increasingly complex array of proteins? Are there shared, overarching signaling pathways which coordinate changing protein expression patterns? We believe that changing physical stress experienced by the myocardium is at least one such signal. Indeed, when viewed from a mechanosensatory viewpoint we can see that many of the proposed t-tubule regulators can be functionally clustered together. For example, several of these proteins have proposed roles in mechanosensing at the cell membrane (PKC *via* stretch-activated channels) or Z-disks (T-Cap), or are linked to the mechanosensitive process of actin polymerization (BIN1, Nexilin, and PKC). A perhaps somewhat distinct group of t-tubule regulatory proteins appears to be focused on maintenance of the membrane itself, and notably includes Mtm1, BIN1, and the general autophagic and endocytic processes. It is less clear that these processes are mechanosensitive in cardiomyocytes, and we might rather speculate that the changing metabolic environment of the diseased heart is an overarching signal that regulates membrane integrity.

Despite promising results from rodent studies, it seems unlikely that targeting of only a single t-tubule regulator would be sufficient to therapeutically protect t-tubule structure in humans. Rather, we believe that new interventions should instead be aimed at the overarching signals and functional groups of proteins described above. For example, upregulating expression of JPH2 has been suggested as an approach to maintain dyadic structure during HFrEF (Reynolds et al., 2016). However, we expect that this approach would yield only temporary benefits since JPH2 downregulation is itself driven by elevated workload (Frisk et al., 2016), and thus the persistence of these mechanical cues would continue to signal detrimental changes in JPH2 and other mechanosensitive proteins. Indeed, it is notable that existing therapeutics for HFrEF act largely to alleviate cardiac workload, and thus are expected to normalize expression of a spectrum of key mechanosensitive t-tubule regulators. As an alternative approach, we speculate that more precisely interrupting the processes of mechanosensation at the levels of the cardiomyocyte membrane and/or cytoskeleton could be broadly beneficial in the treatment of HFrEF patients.

## Conclusion and Summary

This review has summarized our growing appreciation for the role of t-tubules and dyads as critical regulators of cardiomyocyte Ca^2+^ homeostasis, and thus systolic and diastolic function of the heart as a working organ. The discussion has highlighted a consensus view that t-tubule remodeling is a key mechanism contributing to disrupted Ca^2+^ handling and contractility in HFrEF, and a likely contributor to arrythmogenesis, but that distinct forms of remodeling occur during HFpEF. We have described a wealth of evidence indicating key roles of dyadic proteins JPH2, BIN1, and Cav-3, but also newer players and signaling pathways which hold promise. We believe that several current HFrEF therapies preserve t-tubule structure and cardiac function by normalizing expression of these proteins, and that this involves relief of the high workload that drives dyadic disruption. We anticipate that improved safeguarding of t-tubule integrity will serve as a basis for future HFrEF therapy.

## Author Contributions

All authors contributed to the writing of the article and development of the figures.

## Conflict of Interest

The authors declare that the research was conducted in the absence of any commercial or financial relationships that could be construed as a potential conflict of interest.

## Publisher’s Note

All claims expressed in this article are solely those of the authors and do not necessarily represent those of their affiliated organizations, or those of the publisher, the editors and the reviewers. Any product that may be evaluated in this article, or claim that may be made by its manufacturer, is not guaranteed or endorsed by the publisher.

## Abbreviations

CICR: Ca^2+^-induced Ca^2+^ release
Cav-3: Caveolin-3
CRU: Ca^2+^ release unit
DAD: Delayed afterdepolarization
EAD: Early afterdepolarization
EC coupling: Excitation-contraction coupling
HF: Heart failure
HFpEF: Heart failure with preserved ejection fraction
HFrEF: Heart failure with reduced ejection fraction
JPH2: Junctophilin-2
LTCC: L-type Ca^2+^ channel
miR-24: microRNA-24
MG53: Mitsugumin 53
NEXN: Nexilin
NCX: Sodium-calcium exchanger 1
PKC: Protein kinase C
RyR: Ryanodine Receptor
SR: Sarcoplasmic Reticulum
Tcap: Titin cap/Telethonin.

## References

[B1] Agrawal P. B. Pierson C. R. Joshi M. Liu X. Ravenscroft G. Moghadaszadeh B. (2014). SPEG interacts with myotubularin, and its deficiency causes centronuclear myopathy with dilated cardiomyopathy. *Am. J. Hum. Genet.* 95 218–226. 10.1016/j.ajhg.2014.07.004 25087613 PMC4129406

[B2] Al-Qusairi L. Laporte J. (2011). T-tubule biogenesis and triad formation in skeletal muscle and implication in human diseases. *Skelet. Muscle* 1:26. 10.1186/2044-5040-1-26 21797990 PMC3156648

[B3] Arora R. Aistrup G. L. Supple S. Frank C. Singh J. Tai S. (2017). Regional distribution of T-tubule density in left and right atria in dogs. *Heart Rhythm* 14 273–281. 10.1016/j.hrthm.2016.09.022 27670628 PMC5484147

[B4] Baddeley D. Jayasinghe I. D. Lam L. Rossberger S. Cannell M. B. Soeller C. (2009). Optical single-channel resolution imaging of the ryanodine receptor distribution in rat cardiac myocytes. *Proc. Natl. Acad. Sci. U.S.A.* 106 22275–22280. 10.1073/pnas.0908971106 20018773 PMC2799702

[B5] Balijepalli R. C. Lokuta A. J. Maertz N. A. Buck J. M. Haworth R. A. Valdivia H. H. (2003). Depletion of T-tubules and specific subcellular changes in sarcolemmal proteins in tachycardia-induced heart failure. *Cardiovasc. Res.* 59 67–77. 12829177 10.1016/s0008-6363(03)00325-0

[B6] Beavers D. L. Landstrom A. P. Chiang D. Y. Wehrens X. H. (2014). Emerging roles of junctophilin-2 in the heart and implications for cardiac diseases. *Cardiovasc. Res.* 103 198–205. 10.1093/cvr/cvu151 24935431 PMC4809974

[B7] Berliner D. Bauersachs J. (2017). Current drug therapy in chronic heart failure: the new guidelines of the European society of cardiology (ESC). *Korean Circ. J.* 47 543–554. 10.4070/kcj.2017.0030 28955380 PMC5614938

[B8] Bers D. M. (2002). Cardiac excitation-contraction coupling. *Nature* 415 198–205. 11805843 10.1038/415198a

[B9] Biesmans L. Macquaide N. Heinzel F. R. Bito V. Smith G. L. Sipido K. R. (2011). Subcellular heterogeneity of ryanodine receptor properties in ventricular myocytes with low T-tubule density. *PLoS One* 6:e25100. 10.1371/journal.pone.0025100 22022376 PMC3192718

[B10] Brand T. (2003). Heart development: molecular insights into cardiac specification and early morphogenesis. *Dev. Biol.* 258 1–19. 10.1016/s0012-1606(03)00112-x 12781678

[B11] Brette F. Despa S. Bers D. M. Orchard C. H. (2005). Spatiotemporal characteristics of SR Ca^2+^ uptake and release in detubulated rat ventricular myocytes. *J. Mol. Cell. Cardiol.* 39 804–812. 10.1016/j.yjmcc.2005.08.005 16198369

[B12] Brook W. H. Connell S. Cannata J. Maloney J. E. Walker A. M. (1983). Ultrastructure of the myocardium during development from early fetal life to adult life in sheep. *J. Anat.* 137(Pt 4) 729–741. 6668250 PMC1171875

[B13] Bryant S. M. Kong C. H. T. Watson J. J. Gadeberg H. C. James A. F. Cannell M. B. (2018a). Caveolin 3-dependent loss of t-tubular I_Ca_ during hypertrophy and heart failure in mice. *Exp. Physiol.* 103 652–665. 10.1113/ep086731 29473235 PMC6099270

[B14] Bryant S. M. Kong C. H. T. Watson J. J. Gadeberg H. C. Roth D. M. Patel H. H. (2018b). Caveolin-3 KO disrupts t-tubule structure and decreases t-tubular I_Ca_ density in mouse ventricular myocytes. *Am. J. Physiol. Heart Circ. Physiol.* 315 H1101–H1111. 30028203 10.1152/ajpheart.00209.2018 PMC6415741

[B15] Bryant S. M. Kong C. H. Watson J. Cannell M. B. James A. F. Orchard C. H. (2015). Altered distribution of I_Ca_ impairs Ca release at the t-tubules of ventricular myocytes from failing hearts. *J. Mol. Cell. Cardiol.* 86 23–31. 10.1016/j.yjmcc.2015.06.012 26103619 PMC4564288

[B16] Caldwell J. L. Smith C. E. Taylor R. F. Kitmitto A. Eisner D. A. Dibb K. M. (2014). Dependence of cardiac transverse tubules on the BAR domain protein amphiphysin II (BIN-1). *Circ. Res.* 115 986–996. 10.1161/circresaha.116.303448 25332206 PMC4274343

[B17] Cannell M. B. Crossman D. J. Soeller C. (2006). Effect of changes in action potential spike configuration, junctional sarcoplasmic reticulum micro-architecture and altered t-tubule structure in human heart failure. *J. Muscle Res. Cell Motil.* 27 297–306. 10.1007/s10974-006-9089-y 16897575

[B18] Chen B. Guo A. Zhang C. Chen R. Zhu Y. Hong J. (2013). Critical roles of junctophilin-2 in T-tubule and excitation-contraction coupling maturation during postnatal development. *Cardiovasc. Res.* 100 54–62. 10.1093/cvr/cvt180 23860812 PMC3778961

[B19] Chen B. Li Y. Jiang S. Xie Y. P. Guo A. Kutschke W. (2012). beta-Adrenergic receptor antagonists ameliorate myocyte T-tubule remodeling following myocardial infarction. *FASEB J.* 26 2531–2537. 10.1096/fj.11-199505 22375019 PMC3360148

[B20] Chen C. Y. Caporizzo M. A. Bedi K. Vite A. Bogush A. I. Robison P. (2018). Suppression of detyrosinated microtubules improves cardiomyocyte function in human heart failure. *Nat. Med.* 24 1225–1233. 10.1038/s41591-018-0046-2 29892068 PMC6195768

[B21] Cheng H. Lederer M. R. Lederer W. J. Cannell M. B. (1996). Calcium sparks and [Ca^2+^ ]_i_ waves in cardiac myocytes. *Am. J. Physiol.* 270 C148–C159. 8772440 10.1152/ajpcell.1996.270.1.C148

[B22] Cohn J. N. (1996). The management of chronic heart failure. *N. Engl. J. Med.* 335 490–498. 8672155 10.1056/NEJM199608153350707

[B23] Cooper S. T. McNeil P. L. (2015). Membrane repair: mechanisms and pathophysiology. *Physiol. Rev.* 95 1205–1240. 10.1152/physrev.00037.2014 26336031 PMC4600952

[B24] Crocini C. Coppini R. Ferrantini C. Yan P. Loew L. M. Tesi C. (2014). Defects in T-tubular electrical activity underlie local alterations of calcium release in heart failure. *Proc. Natl. Acad. Sci. U.S.A.* 111 15196–15201. 10.1073/pnas.1411557111 25288764 PMC4210349

[B25] Crossman D. J. Shen X. Jullig M. Munro M. Hou Y. Middleditch M. (2017). Increased collagen within the transverse tubules in human heart failure. *Cardiovasc. Res.* 113 879–891. 10.1093/cvr/cvx055 28444133

[B26] Crossman D. J. Young A. A. Ruygrok P. N. Nason G. P. Baddelely D. Soeller C. (2015). T-tubule disease: relationship between t-tubule organization and regional contractile performance in human dilated cardiomyopathy. *J. Mol. Cell. Cardiol.* 84 170–178. 10.1016/j.yjmcc.2015.04.022 25953258 PMC4467993

[B27] Curl C. L. Danes V. R. Bell J. R. Raaijmakers A. J. A. Ip W. T. K. Chandramouli C. (2018). Cardiomyocyte functional etiology in heart failure with preserved ejection fraction is distinctive-a new preclinical model. *J. Am. Heart Assoc.* 7:e007451. 10.1161/JAHA.117.007451 PMC6015350 29858360

[B28] de Diego C. Chen F. Xie L. H. Dave A. S. Thu M. Rongey C. (2008). Cardiac alternans in embryonic mouse ventricles. *Am. J. Physiol. Heart Circ. Physiol.* 294 H433–H440. 18024542 10.1152/ajpheart.01165.2007

[B29] De La Mata A. Tajada S. O’Dwyer S. Matsumoto C. Dixon R. E. Hariharan N. (2019). BIN1 induces the formation of T-Tubules and adult-like Ca^2+^ release units in developing cardiomyocytes. *Stem Cells* 37 54–64. 10.1002/stem.2927 30353632 PMC6312737

[B30] Dibb K. M. Clarke J. D. Horn M. A. Richards M. A. Graham H. K. Eisner D. A. Trafford A. W. (2009). Characterization of an extensive transverse tubular network in sheep atrial myocytes and its depletion in heart failure. *Circ. Heart Fail.* 2 482–489. 10.1161/circheartfailure.109.852228 19808379

[B31] Dixon R. E. Moreno C. M. Yuan C. Opitz-Araya X. Binder M. D. Navedo M. F. (2015). Graded Ca^2+^ /calmodulin-dependent coupling of voltage-gated CaV1.2 channels. *eLife* 4:e05608. 10.7554/eLife.05608 PMC4360655 25714924

[B32] Dostal D. E. Feng H. Nizamutdinov D. Golden H. B. Afroze S. H. Dostal J. D. (2014). Mechanosensing and regulation of cardiac function. *J. Clin. Exp. Cardiol.* 5:314. 10.4172/2155-9880.1000314 PMC4255974 25485172

[B33] Dridi H. Kushnir A. Zalk R. Yuan Q. Melville Z. Marks A. R. (2020). Intracellular calcium leak in heart failure and atrial fibrillation: a unifying mechanism and therapeutic target. *Nat. Rev. Cardiol.* 17 732–747. 10.1038/s41569-020-0394-8 32555383 PMC8362847

[B34] Dyachenko V. Husse B. Rueckschloss U. Isenberg G. (2009). Mechanical deformation of ventricular myocytes modulates both TRPC6 and Kir2.3 channels. *Cell Calcium* 45 38–54. 10.1016/j.ceca.2008.06.003 18635261

[B35] Edwards A. G. Louch W. E. (2017). Species-dependent mechanisms of cardiac arrhythmia: a cellular focus. *Clin. Med. Insights Cardiol.* 1:1179546816686061. 10.1177/1179546816686061 PMC5392019 28469490

[B36] Fabiato A. (1983). Calcium-induced release of calcium from the cardiac sarcoplasmic reticulum. *Am. J. Physiol.* 245 C1–C14. 6346892 10.1152/ajpcell.1983.245.1.C1

[B37] Feridooni H. A. Dibb K. M. Howlett S. E. (2015). How cardiomyocyte excitation, calcium release and contraction become altered with age. *J. Mol. Cell. Cardiol.* 83 62–72. 10.1016/j.yjmcc.2014.12.004 25498213

[B38] Fiegle D. J. Schober M. Dittrich S. Cesnjevar R. Klingel K. Volk T. (2020). Severe T-System remodeling in pediatric viral myocarditis. *Front. Cardiovasc. Med.* 7:624776. 10.3389/fcvm.2020.624776 33537349 PMC7848076

[B39] Forbes M. S. Sperelakis N. (1976). The presence of transverse and axial tubules in the ventricular myocardium of embryonic and neonatal guinea pigs. *Cell Tissue Res.* 166 83–90. 10.1007/bf00215127 942885

[B40] Forsgren S. Thornell L. E. (1981). The development of Purkinje fibres and ordinary myocytes in the bovine fetal heart. An ultrastructural study. *Anat. Embryol.* 162 127–136. 10.1007/bf00306485 7283176

[B41] Forssmann W. G. Girardier L. (1970). A study of the T system in rat heart. *J. Cell Biol.* 44 1–19. 10.1083/jcb.44.1.1 4901374 PMC2107783

[B42] Franzini-Armstrong C. Protasi F. Tijskens P. (2005). The assembly of calcium release units in cardiac muscle. *Ann. N. Y. Acad. Sci.* 1047 76–85. 10.1196/annals.1341.007 16093486

[B43] Frisk M. Koivumaki J. T. Norseng P. A. Maleckar M. M. Sejersted O. M. Louch W. E. (2014). Variable t-tubule organization and Ca^2+^ homeostasis across the atria. *Am. J. Physiol. Heart Circ. Physiol.* 307 H609–H620. 24951751 10.1152/ajpheart.00295.2014

[B44] Frisk M. Le C. Shen X. Roe A. T. Hou Y. Manfra O. (2021). Etiology-dependent impairment of diastolic cardiomyocyte calcium homeostasis in heart failure with preserved ejection fraction. *J. Am. Coll. Cardiol.* 77 405–419. 33509397 10.1016/j.jacc.2020.11.044 PMC7840890

[B45] Frisk M. Ruud M. Espe E. K. Aronsen J. M. Roe A. T. Zhang L. (2016). Elevated ventricular wall stress disrupts cardiomyocyte t-tubule structure and calcium homeostasis. *Cardiovasc. Res.* 112 443–451. 10.1093/cvr/cvw111 27226008 PMC5031949

[B46] Fu Y. Shaw S. A. Naami R. Vuong C. L. Basheer W. A. Guo X. (2016). Isoproterenol promotes rapid ryanodine receptor movement to bridging integrator 1 (BIN1)-organized dyads. *Circulation* 133 388–397. 10.1161/circulationaha.115.018535 26733606 PMC4729615

[B47] Gadeberg H. C. Bond R. C. Kong C. H. Chanoit G. P. Ascione R. Cannell M. B. (2016). Heterogeneity of T-Tubules in pig hearts. *PLoS One* 11:e0156862. 10.1371/journal.pone.0156862 27281038 PMC4900646

[B48] Gaeta S. A. Bub G. Abbott G. W. Christini D. J. (2009). Dynamical mechanism for subcellular alternans in cardiac myocytes. *Circ. Res.* 105 335–342. 10.1161/circresaha.109.197590 19628792 PMC2740370

[B49] Gibbs E. M. Davidson A. E. Telfer W. R. Feldman E. L. Dowling J. J. (2014). The myopathy-causing mutation DNM2-S619L leads to defective tubulation in vitro and in developing zebrafish. *Dis. Model. Mech.* 7 157–161. 24135484 10.1242/dmm.012286 PMC3882057

[B50] Glukhov A. V. Balycheva M. Sanchez-Alonso J. L. Ilkan Z. Alvarez-Laviada A. Bhogal N. (2015). Direct evidence for microdomain-specific localization and remodeling of functional L-Type calcium channels in rat and human atrial myocytes. *Circulation* 132 2372–2384. 10.1161/circulationaha.115.018131 26450916 PMC4689179

[B51] Gonzalez-Jamett A. M. Momboisse F. Haro-Acuna V. Bevilacqua J. A. Caviedes P. Cardenas A. M. (2013). Dynamin-2 function and dysfunction along the secretory pathway. *Front. Endocrinol. (Lausanne)* 4:126. 10.3389/fendo.2013.00126 24065954 PMC3776141

[B52] Gotoh T. (1983). Quantitative studies on the ultrastructural differentiation and growth of mammalian cardiac muscle cells. The atria and ventricles of the cat. *Acta Anat.* 115 168–177. 10.1159/000145687 6837261

[B53] Grandy S. A. Howlett S. E. (2006). Cardiac excitation-contraction coupling is altered in myocytes from aged male mice but not in cells from aged female mice. *Am. J. Physiol. Heart Circ. Physiol.* 291 H2362–H2370. 16731653 10.1152/ajpheart.00070.2006

[B54] Gross P. Johnson J. Romero C. M. Eaton D. M. Poulet C. Sanchez-Alonso J. (2021). Houser, interaction of the joining region in junctophilin-2 with the L-Type Ca^2+^ channel is pivotal for cardiac dyad assembly and intracellular Ca^2+^ dynamics. *Circ. Res.* 128 92–114. 33092464 10.1161/CIRCRESAHA.119.315715 PMC7790862

[B55] Gu Y. Gorelik J. Spohr H. A. Shevchuk A. Lab M. J. Harding S. E. (2002). High-resolution scanning patch-clamp: new insights into cell function. *FASEB J.* 16 748–750. 10.1096/fj.01-1024fje 11923226

[B56] Guo A. Chen R. Wang Y. Huang C. K. Chen B. Kutschke W. (2018a). Transient activation of PKC results in long-lasting detrimental effects on systolic [Ca^2+^ ]_i_ in cardiomyocytes by altering actin cytoskeletal dynamics and T-tubule integrity. *J. Mol. Cell. Cardiol.* 115 104–114. 10.1016/j.yjmcc.2018.01.003 29307535 PMC5839099

[B57] Guo A. Hall D. Zhang C. Peng T. Miller J. D. Kutschke W. (2015). Molecular determinants of calpain-dependent cleavage of Junctophilin-2 protein in cardiomyocytes. *J. Biol. Chem.* 290 17946–17955. 10.1074/jbc.m115.652396 26063807 PMC4505042

[B58] Guo A. Wang Y. Chen B. Wang Y. Yuan J. Zhang L. (2018b). E-C coupling structural protein junctophilin-2 encodes a stress-adaptive transcription regulator. *Science* 362:eaan3303. 10.1126/science.aan3303 30409805 PMC6336677

[B59] Guo A. Zhang C. Wei S. Chen B. Song L. S. (2013). Emerging mechanisms of T-tubule remodelling in heart failure. *Cardiovasc. Res.* 98 204–215. 10.1093/cvr/cvt020 23393229 PMC3697065

[B60] Guo A. Zhang X. Iyer V. R. Chen B. Zhang C. Kutschke W. J. (2014). Overexpression of junctophilin-2 does not enhance baseline function but attenuates heart failure development after cardiac stress. *Proc. Natl. Acad. Sci. U.S.A.* 111 12240–12245. 10.1073/pnas.1412729111 25092313 PMC4143026

[B61] Haas J. Frese K. S. Peil B. Kloos W. Keller A. Nietsch R. (2015). Atlas of the clinical genetics of human dilated cardiomyopathy. *Eur. Heart J.* 36 1123–1135. 25163546 10.1093/eurheartj/ehu301

[B62] Hamaguchi S. Kawakami Y. Honda Y. Nemoto K. Sano A. Namekata I. (2013). Developmental changes in excitation-contraction mechanisms of the mouse ventricular myocardium as revealed by functional and confocal imaging analyses. *J. Pharmacol. Sci.* 123 167–175. 10.1254/jphs.13099fp 24096881

[B63] Han J. Wu H. Wang Q. Wang S. (2013). Morphogenesis of T-tubules in heart cells: the role of junctophilin-2. *Sci. China Life Sci.* 56 647–652. 10.1007/s11427-013-4490-4 23749380

[B64] Hassel D. Dahme T. Erdmann J. Meder B. Huge A. Stoll M. (2009). Nexilin mutations destabilize cardiac Z-disks and lead to dilated cardiomyopathy. *Nat. Med.* 15 1281–1288. 10.1038/nm.2037 19881492

[B65] Hayashi T. Martone M. E. Yu Z. Thor A. Doi M. Holst M. J. (2009). Three-dimensional electron microscopy reveals new details of membrane systems for Ca^2+^ signaling in the heart. *J. Cell Sci.* 122 1005–1013. 10.1242/jcs.028175 19295127 PMC2720931

[B66] He B. Tang R. H. Weisleder N. Xiao B. Yuan Z. Cai C. (2012). Enhancing muscle membrane repair by gene delivery of MG53 ameliorates muscular dystrophy and heart failure in delta-Sarcoglycan-deficient hamsters. *Mol. Ther.* 20 727–735. 10.1038/mt.2012.5 22314291 PMC3321592

[B67] He J. Conklin M. W. Foell J. D. Wolff M. R. Haworth R. A. Coronado R. (2001). Reduction in density of transverse tubules and L-type Ca^2+^ channels in canine tachycardia-induced heart failure. *Cardiovasc. Res.* 49 298–307. 10.1016/s0008-6363(00)00256-x 11164840

[B68] Heinzel F. R. Bito V. Biesmans L. Wu M. Detre E. von Wegner F. (2008). Remodeling of T-tubules and reduced synchrony of Ca^2+^ release in myocytes from chronically ischemic myocardium. *Circ. Res.* 102 338–346. 10.1161/circresaha.107.160085 18079411

[B69] Heinzel F. R. Bito V. Volders P. G. Antoons G. Mubagwa K. Sipido K. R. (2002). Spatial and temporal inhomogeneities during Ca^2+^ release from the sarcoplasmic reticulum in pig ventricular myocytes. *Circ. Res.* 91 1023–1030. 10.1161/01.res.0000045940.67060.dd 12456488

[B70] Henderson S. A. Goldhaber J. I. So J. M. Han T. Motter C. Ngo A. (2004). Functional adult myocardium in the absence of Na^+^-Ca^2+^ exchange: cardiac-specific knockout of NCX1. *Circ. Res.* 95 604–611. 10.1161/01.res.0000142316.08250.68 15308581

[B71] Herring N. Kalla M. Paterson D. J. (2019). The autonomic nervous system and cardiac arrhythmias: current concepts and emerging therapies. *Nat. Rev. Cardiol.* 16 707–726. 10.1038/s41569-019-0221-2 31197232

[B72] Hirakow R. Gotoh T. Watanabe T. (1980). Quantitative studies on the ultrastructural differentiation and growth of mammalian cardiac muscle cells. I. The atria and ventricles of the rat. *Acta Anat.* 108 144–152. 10.1159/000145293 7405533

[B73] Hitzeman T. C. Xie Y. Zadikany R. H. Nikolova A. P. Baum R. Caldaruse A. M. (2020). cBIN1 score (CS) identifies ambulatory HFrEF patients and predicts cardiovascular events. *Front. Physiol.* 11:503. 10.3389/fphys.2020.00503 32670075 PMC7326053

[B74] Hoerter J. Mazet F. Vassort G. (1981). Perinatal growth of the rabbit cardiac cell: possible implications for the mechanism of relaxation. *J. Mol. Cell. Cardiol.* 13 725–740. 10.1016/0022-2828(81)90255-8 7265262

[B75] Hong T. T. Cogswell R. James C. A. Kang G. Pullinger C. R. Malloy M. J. (2012a). Plasma BIN1 correlates with heart failure and predicts arrhythmia in patients with arrhythmogenic right ventricular cardiomyopathy. *Heart Rhythm* 9 961–967. 10.1016/j.hrthm.2012.01.024 22300662 PMC3349006

[B76] Hong T. T. Smyth J. W. Chu K. Y. Vogan J. M. Fong T. S. Jensen B. C. (2012b). BIN1 is reduced and Cav1.2 trafficking is impaired in human failing cardiomyocytes. *Heart Rhythm* 9 812–820. 10.1016/j.hrthm.2011.11.055 22138472 PMC3306544

[B77] Hong T. T. Smyth J. W. Gao D. Chu K. Y. Vogan J. M. Fong T. S. (2010). BIN1 localizes the L-type calcium channel to cardiac T-tubules. *PLoS Biol.* 8:e1000312. 10.1371/journal.pbio.1000312 20169111 PMC2821894

[B78] Hong T. Shaw R. M. (2017). Cardiac T-Tubule microanatomy and function. *Physiol. Rev.* 97 227–252. 10.1152/physrev.00037.2015 27881552 PMC6151489

[B79] Hong T. Yang H. Zhang S. S. Cho H. C. Kalashnikova M. Sun B. (2014). Cardiac BIN1 folds T-tubule membrane, controlling ion flux and limiting arrhythmia. *Nat. Med.* 20 624–632. 10.1038/nm.3543 24836577 PMC4048325

[B80] Hsu H. C. Chen C. Y. Lee B. C. Chen M. F. (2016). High-fat diet induces cardiomyocyte apoptosis via the inhibition of autophagy. *Eur. J. Nutr.* 55 2245–2254. 10.1007/s00394-015-1034-7 26358164

[B81] Huang C. K. Chen B. Y. Guo A. Chen R. Zhu Y. Q. Kutschke W. (2016). Sildenafil ameliorates left ventricular T-tubule remodeling in a pressure overload-induced murine heart failure model. *Acta Pharmacol. Sin.* 37 473–482. 10.1038/aps.2016.13 26972492 PMC4820805

[B82] Huang C. Y. Peres Moreno Maia-Joca R. Ong C. S. Wilson I. DiSilvestre D. Tomaselli G. F. (2020). Enhancement of human iPSC-derived cardiomyocyte maturation by chemical conditioning in a 3D environment. *J. Mol Cell. Cardiol.* 138 1–11. 10.1016/j.yjmcc.2019.10.001 31655038

[B83] Huxley A. F. Taylor R. E. (1955). Function of Krause’s membrane. *Nature* 176:1068. 10.1038/1761068a0 13272749

[B84] Ibrahim M. Al Masri A. Navaratnarajah M. Siedlecka U. Soppa G. K. Moshkov A. (2010). Prolonged mechanical unloading affects cardiomyocyte excitation-contraction coupling, transverse-tubule structure, and the cell surface. *FASEB J.* 24 3321–3329. 10.1096/fj.10-156638 20430793 PMC2923356

[B85] Ibrahim M. Terracciano C. M. (2013). Reversibility of T-tubule remodelling in heart failure: mechanical load as a dynamic regulator of the T-tubules. *Cardiovasc. Res.* 98 225–232. 10.1093/cvr/cvt016 23345265

[B86] Ibrahim M. Gorelik J. Yacoub M. H. Terracciano C. M. (2011). The structure and function of cardiac t-tubules in health and disease. *Proc. Biol. Sci.* 278 2714–2723. 10.1098/rspb.2011.0624 21697171 PMC3145195

[B87] Ibrahim M. Kukadia P. Siedlecka U. Cartledge J. E. Navaratnarajah M. Tokar S. (2012). Cardiomyocyte Ca^2+^ handling and structure is regulated by degree and duration of mechanical load variation. *J. Cell. Mol. Med.* 16 2910–2918. 10.1111/j.1582-4934.2012.01611.x 22862818 PMC4393719

[B88] Ibrahim M. Nader A. Yacoub M. H. Terracciano C. (2015). Manipulation of sarcoplasmic reticulum Ca^2+^ release in heart failure through mechanical intervention. *J. Physiol.* 593 3253–3259. 10.1113/jp270446 25922157 PMC4553050

[B89] Ibrahim M. Siedlecka U. Buyandelger B. Harada M. Rao C. Moshkov A. (2013). A critical role for Telethonin in regulating t-tubule structure and function in the mammalian heart. *Hum. Mol. Genet.* 22 372–383. 23100327 10.1093/hmg/dds434 PMC3526164

[B90] Ito D. W. Hannigan K. I. Ghosh D. Xu B. Del Villar S. G. Xiang Y. K. (2019). beta-adrenergic-mediated dynamic augmentation of sarcolemmal CaV 1.2 clustering and co-operativity in ventricular myocytes. *J. Physiol.* 597 2139–2162. 10.1113/jp277283 30714156 PMC6462464

[B91] Jaquenod De Giusti C. Blanco P. G. Lamas P. A. Carrizo Velasquez F. Lofeudo J. M. Portiansky E. L. (2019). Carbonic anhydrase II/sodium-proton exchanger 1 metabolon complex in cardiomyopathy of ob(-/-) type 2 diabetic mice. *J. Mol. Cell. Cardiol.* 136 53–63. 10.1016/j.yjmcc.2019.09.005 31518570

[B92] Jayasinghe I. D. Cannell M. B. Soeller C. (2009). Organization of ryanodine receptors, transverse tubules, and sodium-calcium exchanger in rat myocytes. *Biophys. J.* 97 2664–2673. 10.1016/j.bpj.2009.08.036 19917219 PMC2776253

[B93] Jayasinghe I. Clowsley A. H. Lin R. Lutz T. Harrison C. Green E. (2018). True molecular scale visualization of variable clustering properties of ryanodine receptors. *Cell Rep.* 22 557–567. 10.1016/j.celrep.2017.12.045 29320748 PMC5775502

[B94] Jayasinghe I. Crossman D. Soeller C. Cannell M. (2012). Comparison of the organization of T-tubules, sarcoplasmic reticulum and ryanodine receptors in rat and human ventricular myocardium. *Clin. Exp. Pharmacol. Physiol.* 39 469–476. 10.1111/j.1440-1681.2011.05578.x 21790719

[B95] Jiang M. Zhang M. Howren M. Wang Y. Tan A. Balijepalli R. C. (2016). JPH-2 interacts with Ca_i_-handling proteins and ion channels in dyads: contribution to premature ventricular contraction-induced cardiomyopathy. *Heart Rhythm* 13 743–752. 10.1016/j.hrthm.2015.10.037 26538326 PMC4762763

[B96] Jiang Y. Tanaka H. Matsuyama T. A. Yamaoka Y. Takamatsu T. (2014). Pacing-induced non-uniform Ca^2+^ dynamics in rat atria revealed by rapid-scanning confocal microscopy. *Acta Histochem. Cytochem.* 47 59–65. 10.1267/ahc.14014 25221364 PMC4138402

[B97] Jones P. P. MacQuaide N. Louch W. E. (2018). Dyadic plasticity in cardiomyocytes. *Front. Physiol.* 9:1773. 10.3389/fphys.2018.01773 30618792 PMC6298195

[B98] Kaprielian R. R. Stevenson S. Rothery S. M. Cullen M. J. Severs N. J. (2000). Distinct patterns of dystrophin organization in myocyte sarcolemma and transverse tubules of normal and diseased human myocardium. *Circulation* 101 2586–2594. 10.1161/01.cir.101.22.2586 10840009

[B99] Kemi O. J. Hoydal M. A. Macquaide N. Haram P. M. Koch L. G. Britton S. L. (2011). The effect of exercise training on transverse tubules in normal, remodeled, and reverse remodeled hearts. *J. Cell Physiol.* 226 2235–2243. 10.1002/jcp.22559 21660947 PMC3096693

[B100] Ketel K. Krauss M. Nicot A. S. Puchkov D. Wieffer M. Muller R. (2016). A phosphoinositide conversion mechanism for exit from endosomes. *Nature* 529 408–412. 10.1038/nature16516 26760201

[B101] Kilfoil P. J. Lotteau S. Zhang R. Yue X. Aynaszyan S. Solymani R. E. (2020). Distinct features of calcium handling and beta-adrenergic sensitivity in heart failure with preserved versus reduced ejection fraction. *J. Physiol.* 598 5091–5108. 10.1113/jp280425 32829489 PMC7693093

[B102] Kim H. D. Kim D. J. I Lee J. Rah B. J. Sawa Y. Schaper J. (1992). Human fetal heart development after mid-term: morphometry and ultrastructural study. *J. Mol. Cell. Cardiol.* 24 949–965. 10.1016/0022-2828(92)91862-y 1433323

[B103] Kitmitto A. Baudoin F. Cartwright E. J. (2019). Cardiomyocyte damage control in heart failure and the role of the sarcolemma. *J. Muscle Res. Cell Motil.* 40 319–333. 10.1007/s10974-019-09539-5 31520263 PMC6831538

[B104] Kojima C. Hashimoto A. Yabuta I. Hirose M. Hashimoto S. Kanaho Y. (2004). Regulation of Bin1 SH3 domain binding by phosphoinositides. *EMBO J.* 23 4413–4422. 10.1038/sj.emboj.7600442 15483625 PMC526460

[B105] Kolstad T. R. van den Brink J. MacQuaide N. Lunde P. K. Frisk M. Aronsen J. M. (2018). Ryanodine receptor dispersion disrupts Ca^2+^ release in failing cardiac myocytes. *eLife* 7:e39427. 10.7554/eLife.39427 PMC6245731 30375974

[B106] Kong C. H. T. Bryant S. M. Watson J. J. Gadeberg H. C. Roth D. M. Patel H. H. (2018a). The effects of aging on the regulation of T-Tubular I_Ca_ by caveolin in mouse ventricular myocytes. *J. Gerontol. A Biol. Sci. Med. Sci.* 73 711–719. 10.1093/gerona/glx242 29236992 PMC5946816

[B107] Kong C. H. T. Bryant S. M. Watson J. J. Roth D. M. Patel H. H. Cannell M. B. (2019). Cardiac-specific overexpression of caveolin-3 preserves t-tubular I_Ca_ during heart failure in mice. *Exp. Physiol.* 104 654–666. 10.1113/ep087304 30786093 PMC6488395

[B108] Kong C. H. T. Rog-Zielinska E. A. Kohl P. Orchard C. H. Cannell M. B. (2018b). Solute movement in the t-tubule system of rabbit and mouse cardiomyocytes. *Proc. Natl. Acad. Sci. U.S.A.* 115 E7073–E7080. 29991602 10.1073/pnas.1805979115 PMC6065001

[B109] Korhonen T. Rapila R. Ronkainen V. P. Koivumaki J. T. Tavi P. (2010). Local Ca^2+^ releases enable rapid heart rates in developing cardiomyocytes. *J. Physiol.* 588 1407–1417. 10.1113/jphysiol.2009.185173 20211983 PMC2876799

[B110] Kostin S. Scholz D. Shimada T. Maeno Y. Mollnau H. Hein S. (1998). The internal and external protein scaffold of the T-tubular system in cardiomyocytes. *Cell Tissue Res.* 294 449–460. 10.1007/s004410051196 9799462

[B111] Lahiri S. K. Quick A. P. Samson-Couterie B. Hulsurkar M. Elzenaar I. van Oort R. J. (2020). Nuclear localization of a novel calpain-2 mediated junctophilin-2 C-terminal cleavage peptide promotes cardiomyocyte remodeling. *Basic Res. Cardiol.* 115:49. 10.1007/s00395-020-0807-1 PMC10113426 32592107

[B112] Lam C. S. P. Gamble G. D. Ling L. H. Sim D. Leong K. T. G. Yeo P. S. D. (2018). Mortality associated with heart failure with preserved vs. reduced ejection fraction in a prospective international multi-ethnic cohort study. *Eur. Heart J.* 39 1770–1780. 10.1093/eurheartj/ehy005 29390051

[B113] Lambert R. Srodulski S. Peng X. Margulies K. B. Despa F. Despa S. (2015). Intracellular Na^+^ concentration ([Na^+^]_i_) is elevated in diabetic hearts due to enhanced Na^+^-Glucose cotransport. *J. Am. Heart Assoc.* 4:e002183. 10.1161/JAHA.115.002183 PMC4599504 26316524

[B114] Lammerding J. Kamm R. D. Lee R. T. (2004). Mechanotransduction in cardiac myocytes. *Ann. N. Y. Acad. Sci.* 1015 53–70. 10.1196/annals.1302.005 15201149

[B115] Landstrom A. P. Kellen C. A. Dixit S. S. van Oort R. J. Garbino A. Weisleder N. (2011). Junctophilin-2 expression silencing causes cardiocyte hypertrophy and abnormal intracellular calcium-handling. *Circ. Heart Fail.* 4 214–223. 10.1161/circheartfailure.110.958694 21216834 PMC3059380

[B116] Larbig R. Torres N. Bridge J. H. Goldhaber J. I. Philipson K. D. (2010). Activation of reverse Na^+^-Ca^2+^ exchange by the Na^+^ current augments the cardiac Ca^2+^ transient: evidence from NCX knockout mice. *J. Physiol.* 588 3267–3276. 10.1113/jphysiol.2010.187708 20643777 PMC2976021

[B117] Lawless M. Caldwell J. L. Radcliffe E. J. Smith C. E. R. Madders G. W. P. Hutchings D. C. (2019). Phosphodiesterase 5 inhibition improves contractile function and restores transverse tubule loss and catecholamine responsiveness in heart failure. *Sci. Rep.* 9:6801. 10.1038/s41598-019-42592-1 PMC6494852 31043634

[B118] Lee E. Marcucci M. Daniell L. Pypaert M. Weisz O. A. Ochoa G. C. (2002). Amphiphysin 2 (Bin1) and T-tubule biogenesis in muscle. *Science* 297 1193–1196. 10.1126/science.1071362 12183633

[B119] Lekavich C. L. Barksdale D. J. Neelon V. Wu J. R. (2015). Heart failure preserved ejection fraction (HFpEF): an integrated and strategic review. *Heart Fail. Rev.* 20 643–653. 10.1007/s10741-015-9506-7 26404098

[B120] Lenaerts I. Bito V. Heinzel F. R. Driesen R. B. Holemans P. D’Hooge J. (2009). Ultrastructural and functional remodeling of the coupling between Ca^2+^ influx and sarcoplasmic reticulum Ca^2+^ release in right atrial myocytes from experimental persistent atrial fibrillation. *Circ. Res.* 105 876–885. 10.1161/circresaha.109.206276 19762679

[B121] Li J. Agvanian S. Zhou K. Shaw R. M. Hong T. (2020). Exogenous cardiac bridging Integrator 1 benefits mouse hearts with pre-existing pressure overload-induced heart failure. *Front. Physiol.* 11:708. 10.3389/fphys.2020.00708 32670093 PMC7327113

[B122] Li L. L. Guo Q. J. Lou H. Y. Liang J. H. Yang Y. Xing X. (2020). Nanobar array assay revealed complementary roles of BIN1 splice isoforms in cardiac T-Tubule morphogenesis. *Nano Lett.* 20 6387–6395. 10.1021/acs.nanolett.0c01957 32787151 PMC8486496

[B123] Li Q. O’Neill S. C. Tao T. Li Y. Eisner D. Zhang H. (2012). Mechanisms by which cytoplasmic calcium wave propagation and alternans are generated in cardiac atrial myocytes lacking T-tubules-insights from a simulation study. *Biophys. J.* 102 1471–1482. 10.1016/j.bpj.2012.03.007 22500747 PMC3318133

[B124] Li R. C. Tao J. Guo Y. B. Wu H. D. Liu R. F. Bai Y. (2013). In vivo suppression of microRNA-24 prevents the transition toward decompensated hypertrophy in aortic-constricted mice. *Circ. Res.* 112 601–605. 10.1161/circresaha.112.300806 23307820 PMC3622206

[B125] Lichter J. G. Carruth E. Mitchell C. Barth A. S. Aiba T. Kass D. A. (2014). Remodeling of the sarcomeric cytoskeleton in cardiac ventricular myocytes during heart failure and after cardiac resynchronization therapy. *J. Mol. Cell. Cardiol.* 72 186–195. 10.1016/j.yjmcc.2014.03.012 24657727 PMC4077200

[B126] Lindner E. (1957). [Submicroscopic morphology of the cardiac muscle]. *Z. Zellforsch. Mikrosk. Anat. (Vienna, Austria: 1948)* 45 702–746. 13456982

[B127] Lines G. T. Sande J. B. Louch W. E. Mork H. K. Grottum P. Sejersted O. M. (2006). Contribution of the Na^+^/Ca^2+^ exchanger to rapid Ca^2+^ release in cardiomyocytes. *Biophys. J.* 91 779–792. 10.1529/biophysj.105.072447 16679359 PMC1563770

[B128] Linke W. A. (2008). Sense and stretchability: the role of titin and titin-associated proteins in myocardial stress-sensing and mechanical dysfunction. *Cardiovasc. Res.* 77 637–648. 17475230 10.1016/j.cardiores.2007.03.029

[B129] Lipsett D. B. Frisk M. Aronsen J. M. Norden E. S. Buonarati O. R. Cataliotti A. (2019). Cardiomyocyte substructure reverts to an immature phenotype during heart failure. *J. Physiol.* 59 1833–1853. 10.1113/jp277273 30707448 PMC6441900

[B130] Liu C. Spinozzi S. Chen J. Y. Fang X. Feng W. Perkins G. (2019). Nexilin is a new component of junctional membrane complexes required for cardiac T-Tubule formation. *Circulation* 140 55–66. 10.1161/circulationaha.119.039751 30982350 PMC6889818

[B131] Liu C. Spinozzi S. Feng W. Chen Z. Zhang L. Zhu S. (2020). Homozygous G650del nexilin variant causes cardiomyopathy in mice. *JCI Insight* 5:e138780. 10.1172/jci.insight.138780 PMC7455123 32814711

[B132] Liu Y. Zhou K. Li J. Agvanian S. Caldaruse A. M. Shaw S. (2020). In mice subjected to chronic stress, exogenous cBIN1 preserves calcium-handling machinery and cardiac function. *JACC Basic Transl. Sci.* 5 561–578. 10.1016/j.jacbts.2020.03.006 32613144 PMC7315191

[B133] Louch W. E. Nattel S. (2017). T-tubular collagen: a new player in mechanosensing and disease? *Cardiovasc. Res.* 113 839–840. 10.1093/cvr/cvx091 28481973 PMC5852544

[B134] Louch W. E. Bito V. Heinzel F. R. Macianskiene R. Vanhaecke J. Flameng W. (2004). Reduced synchrony of Ca^2+^ release with loss of T-tubules-a comparison to Ca^2+^ release in human failing cardiomyocytes. *Cardiovasc. Res.* 62 63–73. 10.1016/j.cardiores.2003.12.031 15023553

[B135] Louch W. E. Hake J. Jolle G. F. Mork H. K. Sjaastad I. Lines G. T. (2010a). Control of Ca^2+^ release by action potential configuration in normal and failing murine cardiomyocytes. *Biophys. J.* 99 1377–1386. 10.1016/j.bpj.2010.06.055 20816049 PMC2931738

[B136] Louch W. E. Hake J. Mork H. K. Hougen K. Skrbic B. Ursu D. (2013). Slow Ca^2+^ sparks de-synchronize Ca^2+^ release in failing cardiomyocytes: evidence for altered configuration of Ca^2+^ release units? *J. Mol. Cell. Cardiol.* 58 41–52. 10.1016/j.yjmcc.2013.01.014 23376034

[B137] Louch W. E. Koivumaki J. T. Tavi P. (2015). Calcium signalling in developing cardiomyocytes: implications for model systems and disease. *J. Physiol.* 593 1047–1063. 10.1113/jphysiol.2014.274712 25641733 PMC4358669

[B138] Louch W. E. Mork H. K. Sexton J. Stromme T. A. Laake P. Sjaastad I. (2006). T-tubule disorganization and reduced synchrony of Ca^2+^ release in murine cardiomyocytes following myocardial infarction. *J. Physiol.* 574 519–533. 10.1113/jphysiol.2006.107227 16709642 PMC1817777

[B139] Louch W. E. Sejersted O. M. Swift F. (2010b). There goes the neighborhood: pathological alterations in T-tubule morphology and consequences for cardiomyocyte Ca^2+^ handling. *J. Biomed. Biotechnol.* 2010:503906. 10.1155/2010/503906 PMC2852607 20396394

[B140] Louch W. E. Stokke M. K. Sjaastad I. Christensen G. Sejersted O. M. (2012). No rest for the weary: diastolic calcium homeostasis in the normal and failing myocardium. *Physiology (Bethesda)* 27 308–323. 10.1152/physiol.00021.2012 23026754

[B141] Loucks A. D. O’Hara T. Trayanova N. A. (2018). Degradation of T-Tubular microdomains and altered cAMP compartmentation lead to emergence of arrhythmogenic triggers in heart failure myocytes: an in silico study. *Front. Physiol.* 9:1737. 10.3389/fphys.2018.01737 30564142 PMC6288429

[B142] Lyon A. R. MacLeod K. T. Zhang Y. Garcia E. Kanda G. K. Lab M. J. (2009). Loss of T-tubules and other changes to surface topography in ventricular myocytes from failing human and rat heart. *Proc. Natl. Acad. Sci. U.S.A.* 106 6854–6859. 10.1073/pnas.0809777106 19342485 PMC2672472

[B143] Lyon A. R. Nikolaev V. O. Miragoli M. Sikkel M. B. Paur H. Benard L. (2012). Plasticity of surface structures and beta(2)-adrenergic receptor localization in failing ventricular cardiomyocytes during recovery from heart failure. *Circ. Heart Fail.* 5 357–365. 10.1161/circheartfailure.111.964692 22456061 PMC4886822

[B144] Lyu Y. Verma V. K. Lee Y. Taleb I. Badolia R. Shankar T. S. (2021). Remodeling of t-system and proteins underlying excitation-contraction coupling in aging versus failing human heart. *NPJ Aging Mech. Dis.* 7:16. 10.1038/s41514-021-00066-7 PMC8163749 34050186

[B145] Mackova K. Zahradnikova A. Jr. Hotka M. Hoffmannova B. Zahradnik I. Zahradnikova A. (2017). Calcium release-dependent inactivation precedes formation of the tubular system in developing rat cardiac myocytes. *Eur. Biophys. J.* 46 691–703. 10.1007/s00249-017-1249-z 28913625

[B146] Manfra O. Frisk M. Louch W. E. (2017). Regulation of cardiomyocyte t-tubular structure: opportunities for therapy. *Curr. Heart Fail. Rep.* 14 167–178. 10.1007/s11897-017-0329-9 28447290 PMC5423965

[B147] Maron B. J. Ferrans V. J. Roberts W. C. (1975). Ultrastructural features of degenerated cardiac muscle cells in patients with cardiac hypertrophy. *Am. J. Pathol.* 79 387–434. 124533 PMC1912738

[B148] Marx S. O. Gaburjakova J. Gaburjakova M. Henrikson C. Ondrias K. Marks A. R. (2001). Coupled gating between cardiac calcium release channels (ryanodine receptors). *Circ. Res.* 88 1151–1158. 10.1161/hh1101.091268 11397781

[B149] McNary T. G. Bridge J. H. Sachse F. B. (2011). Strain transfer in ventricular cardiomyocytes to their transverse tubular system revealed by scanning confocal microscopy. *Biophys. J.* 100 L53–L55. 21575564 10.1016/j.bpj.2011.03.046 PMC3093556

[B150] McNutt N. S. (1975). Ultrastructure of the myocardial sarcolemma. *Circ. Res.* 37 1–13. 10.1161/01.res.37.1.1 1149179

[B151] Meethal S. V. Potter K. T. Redon D. Munoz-del-Rio A. Kamp T. J. Valdivia H. H. (2007). Structure-function relationships of Ca spark activity in normal and failing cardiac myocytes as revealed by flash photography. *Cell Calcium* 41 123–134. 10.1016/j.ceca.2006.05.006 16837043

[B152] Melnyk P. Zhang L. Shrier A. Nattel S. (2002). Differential distribution of Kir2.1 and Kir2.3 subunits in canine atrium and ventricle. *Am. J. Physiol. Heart Circ. Physiol.* 283 H1123–H1133. 12181143 10.1152/ajpheart.00934.2001

[B153] Minamisawa S. Oshikawa J. Takeshima H. Hoshijima M. Wang Y. Chien K. R. (2004). Junctophilin type 2 is associated with caveolin-3 and is down-regulated in the hypertrophic and dilated cardiomyopathies. *Biochem. Biophys. Res. Commun.* 325 852–856. 10.1016/j.bbrc.2004.10.107 15541368

[B154] Mohler P. J. Davis J. Q. Bennett V. (2005). Ankyrin-B coordinates the Na/K ATPase, Na/Ca exchanger, and InsP_3_ receptor in a cardiac T-tubule/SR microdomain. *PLoS Biol.* 3:e423. 10.1371/journal.pbio.0030423 16292983 PMC1287507

[B155] Mork H. K. Sjaastad I. Sande J. B. Periasamy M. Sejersted O. M. Louch W. E. (2007). Increased cardiomyocyte function and Ca^2+^ transients in mice during early congestive heart failure. *J. Mol. Cell. Cardiol.* 43 177–186. 10.1016/j.yjmcc.2007.05.004 17574269

[B156] Muller A. J. Baker J. F. DuHadaway J. B. Ge K. Farmer G. Donover P. S. (2003). Targeted disruption of the murine Bin1/Amphiphysin II gene does not disable endocytosis but results in embryonic cardiomyopathy with aberrant myofibril formation. *Mol. Cell. Biol.* 23 4295–4306. 10.1128/mcb.23.12.4295-4306.2003 12773571 PMC156129

[B157] Munro M. L. I Jayasinghe D. Wang Q. Quick A. Wang W. Baddeley D. (2016). Junctophilin-2 in the nanoscale organisation and functional signalling of ryanodine receptor clusters in cardiomyocytes. *J. Cell Sci.* 129 4388–4398. 27802169 10.1242/jcs.196873 PMC5201013

[B158] Munro M. L. Soeller C. (2016). Early transverse tubule development begins in utero in the sheep heart. *J. Muscle Res. Cell. Motil.* 37 195–202. 10.1007/s10974-016-9462-4 28062939

[B159] Nikolova A. P. Hitzeman T. C. Baum R. Caldaruse A. M. Agvanian S. Xie Y. (2018). Association of a novel diagnostic biomarker, the plasma cardiac bridging Integrator 1 score, with heart failure with preserved ejection fraction and cardiovascular hospitalization. *JAMA Cardiol.* 3 1206–1210. 10.1001/jamacardio.2018.3539 30383171 PMC6583707

[B160] Nishi M. Mizushima A. Nakagawara K. Takeshima H. (2000). Characterization of human junctophilin subtype genes. *Biochem. Biophys. Res. Commun.* 273 920–927. 10.1006/bbrc.2000.3011 10891348

[B161] Nyström G. (1897). Über die lymphbahnen des Herzens. *Arch. Anat. Physiol. (Anat. Abt.)* 12 361–378.

[B162] Oakley R. H. Ren R. Cruz-Topete D. Bird G. S. Myers P. H. Boyle M. C. (2013). Essential role of stress hormone signaling in cardiomyocytes for the prevention of heart disease. *Proc. Natl. Acad. Sci. U.S.A.* 110 17035–17040. 10.1073/pnas.1302546110 24082121 PMC3801058

[B163] Orchard C. H. Bryant S. M. James A. F. (2013). Do t-tubules play a role in arrhythmogenesis in cardiac ventricular myocytes? *J. Physiol.* 591 4141–4147. 10.1113/jphysiol.2013.254540 23652596 PMC3779108

[B164] Oyehaug L. Loose K. O. Jolle G. F. Roe A. T. Sjaastad I. Christensen G. (2013). Synchrony of cardiomyocyte Ca^2+^ release is controlled by T-tubule organization, SR Ca^2+^ content, and ryanodine receptor Ca^2+^ sensitivity. *Biophys. J.* 104 1685–1697. 10.1016/j.bpj.2013.03.022 23601316 PMC3627865

[B165] Parikh S. S. Blackwell D. J. Gomez-Hurtado N. Frisk M. Wang L. Kim K. (2017). Thyroid and glucocorticoid hormones promote functional t-tubule development in human-induced pluripotent stem cell-derived cardiomyocytes. *Circ. Res.* 121 1323–1330. 10.1161/circresaha.117.311920 28974554 PMC5722667

[B166] Parton R. G. Way M. Zorzi N. Stang E. (1997). Caveolin-3 associates with developing T-tubules during muscle differentiation. *J. Cell Biol.* 136 137–154. 10.1083/jcb.136.1.137 9008709 PMC2132459

[B167] Paulus W. J. Tschope C. (2013). A novel paradigm for heart failure with preserved ejection fraction: comorbidities drive myocardial dysfunction and remodeling through coronary microvascular endothelial inflammation. *J. Am. Coll. Cardiol.* 62 263–271. 23684677 10.1016/j.jacc.2013.02.092

[B168] Picas L. Viaud J. Schauer K. Vanni S. Hnia K. Fraisier V. (2014). BIN1/M-Amphiphysin2 induces clustering of phosphoinositides to recruit its downstream partner dynamin. *Nat. Commun.* 5:5647. 10.1038/ncomms6647 25487648

[B169] Pieske B. Tschope C. de Boer R. A. Fraser A. G. Anker S. D. Donal E. (2019). How to diagnose heart failure with preserved ejection fraction: the HFA-PEFF diagnostic algorithm: a consensus recommendation from the Heart Failure Association (HFA) of the European society of cardiology (ESC). *Eur. Heart J.* 40 3297–3317. 10.1093/eurheartj/ehz641 31504452

[B170] Pinali C. Bennett H. Davenport J. B. Trafford A. W. Kitmitto A. (2013). Three-dimensional reconstruction of cardiac sarcoplasmic reticulum reveals a continuous network linking transverse-tubules: this organization is perturbed in heart failure. *Circ. Res.* 113 1219–1230. 10.1161/circresaha.113.301348 24044951

[B171] Pinali C. Malik N. Davenport J. B. Allan L. J. Murfitt L. Iqbal M. M. (2017). Post-myocardial infarction T-tubules form enlarged branched structures with dysregulation of Junctophilin-2 and Bridging Integrator 1 (BIN-1). *J. Am. Heart Assoc.* 6:e004834. 10.1161/JAHA.116.004834 PMC5524063 28473402

[B172] Prins K. W. Asp M. L. Zhang H. Wang W. Metzger J. M. (2016). Microtubule-mediated misregulation of Junctophilin-2 underlies T-Tubule disruptions and calcium mishandling in mdx Mice. *JACC Basic Transl. Sci.* 1 122–130. 10.1016/j.jacbts.2016.02.002 27482548 PMC4965806

[B173] Prokic I. Cowling B. S. Kutchukian C. Kretz C. Tasfaout H. Gache V. (2020). Differential physiological roles for BIN1 isoforms in skeletal muscle development, function and regeneration. *Dis. Model. Mech.* 13:dmm044354. 10.1242/dmm.044354 PMC7710016 32994313

[B174] Quick A. P. Wang Q. Philippen L. E. Barreto-Torres G. Chiang D. Y. Beavers D. (2017). SPEG (Striated Muscle Preferentially Expressed Protein Kinase) is essential for cardiac function by regulating junctional membrane complex activity. *Circ. Res.* 120 110–119. 10.1161/circresaha.116.309977 27729468 PMC5218854

[B175] Rajabi M. Kassiotis C. Razeghi P. Taegtmeyer H. (2007). Return to the fetal gene program protects the stressed heart: a strong hypothesis. *Heart Fail. Rev.* 12 331–343. 10.1007/s10741-007-9034-1 17516164

[B176] Rapila R. Korhonen T. Tavi P. (2008). Excitation-contraction coupling of the mouse embryonic cardiomyocyte. *J. Gen. Physiol.* 132 397–405. 10.1085/jgp.200809960 18794377 PMC2553387

[B177] Retzius G. (1881). Zur Kenntnis der quergestreiften Muskelfaser. *Biol. Untersuch.* 1 1–26.

[B178] Reynolds J. O. Chiang D. Y. Wang W. Beavers D. L. Dixit S. S. Skapura D. G. (2013). Junctophilin-2 is necessary for T-tubule maturation during mouse heart development. *Cardiovasc. Res.* 100 44–53. 10.1093/cvr/cvt133 23715556 PMC3778955

[B179] Reynolds J. O. Quick A. P. Wang Q. Beavers D. L. Philippen L. E. Showell J. (2016). Junctophilin-2 gene therapy rescues heart failure by normalizing RyR2-mediated Ca^2+^ release. *Int. J. Cardiol.* 225 371–380. 10.1016/j.ijcard.2016.10.021 27760414 PMC5101129

[B180] Richards M. A. Clarke J. D. Saravanan P. Voigt N. Dobrev D. Eisner D. A. (2011). Transverse tubules are a common feature in large mammalian atrial myocytes including human. *Am. J. Physiol. Heart Circ. Physiol.* 301 H1996–H2005. 21841013 10.1152/ajpheart.00284.2011 PMC3213978

[B181] Roe A. T. Aronsen J. M. Skardal K. Hamdani N. Linke W. A. Danielsen H. E. (2017). Increased passive stiffness promotes diastolic dysfunction despite improved Ca^2+^ handling during left ventricular concentric hypertrophy. *Cardiovasc. Res.* 113 1161–1172. 10.1093/cvr/cvx087 28472418 PMC5852536

[B182] Roe A. T. Frisk M. Louch W. E. (2015). Targeting cardiomyocyte Ca^2+^ homeostasis in heart failure. *Curr. Pharm. Des.* 21 431–448. 10.2174/138161282104141204124129 25483944 PMC4475738

[B183] Roe A. T. Ruud M. Espe E. K. Manfra O. Longobardi S. Aronsen J. M. (2019). Regional diastolic dysfunction in post-infarction heart failure: role of local mechanical load and SERCA expression. *Cardiovasc. Res.* 115 752–764. 10.1093/cvr/cvy257 30351410 PMC6432054

[B184] Rog-Zielinska E. A. Moss R. Kaltenbacher W. Greiner J. Verkade P. Seemann G. (2021a). Nano-scale morphology of cardiomyocyte t-tubule/sarcoplasmic reticulum junctions revealed by ultra-rapid high-pressure freezing and electron tomography. *J. Mol. Cell. Cardiol.* 153 86–92. 10.1016/j.yjmcc.2020.12.006 33359037 PMC8035077

[B185] Rog-Zielinska E. A. Scardigli M. Peyronnet R. Zgierski-Johnston C. M. Greiner J. Madl J. (2021b). Beat-by-Beat cardiomyocyte t-tubule deformation drives tubular content exchange. *Circ. Res.* 128 203–215. 10.1161/circresaha.120.317266 33228470 PMC7834912

[B186] Ronaldson-Bouchard K. Ma S. P. Yeager K. Chen T. Song L. Sirabella D. (2018). Advanced maturation of human cardiac tissue grown from pluripotent stem cells. *Nature* 556 239–243. 10.1038/s41586-018-0016-3 29618819 PMC5895513

[B187] Royer B. Hnia K. Gavriilidis C. Tronchere H. Tosch V. Laporte J. (2013). The myotubularin-amphiphysin 2 complex in membrane tubulation and centronuclear myopathies. *EMBO Rep.* 14 907–915. 10.1038/embor.2013.119 23917616 PMC3807231

[B188] Russell J. Du Toit E. F. Peart J. N. Patel H. H. Headrick J. P. (2017). Myocyte membrane and microdomain modifications in diabetes: determinants of ischemic tolerance and cardioprotection. *Cardiovasc. Diabetol.* 16:155. 10.1186/s12933-017-0638-z PMC5716308 29202762

[B189] Sachse F. B. Torres N. S. Savio-Galimberti E. Aiba T. Kass D. A. Tomaselli G. F. (2012). Subcellular structures and function of myocytes impaired during heart failure are restored by cardiac resynchronization therapy. *Circ. Res.* 110 588–597. 10.1161/circresaha.111.257428 22253411 PMC3299196

[B190] Sah R. Ramirez R. J. Backx P. H. (2002). Modulation of Ca^2+^ release in cardiac myocytes by changes in repolarization rate: role of phase-1 action potential repolarization in excitation-contraction coupling. *Circ. Res.* 90 165–173. 10.1161/hh0202.103315 11834709

[B191] Sanchez-Alonso J. L. Bhargava A. O’Hara T. Glukhov A. V. Schobesberger S. Bhogal N. (2016). Microdomain-specific modulation of L-Type calcium channels leads to triggered ventricular arrhythmia in heart failure. *Circ. Res.* 119 944–955. 10.1161/circresaha.116.308698 27572487 PMC5045818

[B192] Savio-Galimberti E. Frank J. Inoue M. Goldhaber J. I. Cannell M. B. Bridge J. H. (2008). Novel features of the rabbit transverse tubular system revealed by quantitative analysis of three-dimensional reconstructions from confocal images. *Biophys. J.* 95 2053–2062. 10.1529/biophysj.108.130617 18487298 PMC2483780

[B193] Scardigli M. Crocini C. Ferrantini C. Gabbrielli T. Silvestri L. Coppini R. (2017). Quantitative assessment of passive electrical properties of the cardiac T-tubular system by FRAP microscopy. *Proc. Natl. Acad. Sci. U.S.A.* 114 5737–5742. 10.1073/pnas.1702188114 28507142 PMC5465880

[B194] Schaper J. Froede R. Hein S. Buck A. Hashizume H. Speiser B. (1991). Impairment of the myocardial ultrastructure and changes of the cytoskeleton in dilated cardiomyopathy. *Circulation* 83 504–514. 10.1161/01.cir.83.2.504 1991369

[B195] Schmitto J. D. Krabatsch T. Damme L. Netuka I. (2018). Less invasive HeartMate 3 left ventricular assist device implantation. *J. Thorac. Dis.* 10 S1692–S1695. 30034840 10.21037/jtd.2018.01.26 PMC6035962

[B196] Schobesberger S. Wright P. Tokar S. Bhargava A. Mansfield C. Glukhov A. V. (2017). T-tubule remodelling disturbs localized beta2-adrenergic signalling in rat ventricular myocytes during the progression of heart failure. *Cardiovasc. Res.* 113 770–782. 10.1093/cvr/cvx074 28505272 PMC5437368

[B197] Schulson M. N. Scriven D. R. Fletcher P. Moore E. D. (2011). Couplons in rat atria form distinct subgroups defined by their molecular partners. *J. Cell Sci.* 124 1167–1174. 10.1242/jcs.080929 21385843 PMC3056609

[B198] Scriven D. R. Asghari P. Schulson M. N. Moore E. D. (2010). Analysis of Cav1.2 and ryanodine receptor clusters in rat ventricular myocytes. *Biophys. J.* 99 3923–3929. 10.1016/j.bpj.2010.11.008 21156134 PMC3000512

[B199] Scriven D. R. Dan P. Moore E. D. (2000). Distribution of proteins implicated in excitation-contraction coupling in rat ventricular myocytes. *Biophys. J.* 79 2682–2691. 10.1016/s0006-3495(00)76506-4 11053140 PMC1301148

[B200] Seidel T. Fiegle D. J. Baur T. J. Ritzer A. Nay S. Heim C. (2019). Glucocorticoids preserve the t-tubular system in ventricular cardiomyocytes by upregulation of autophagic flux. *Basic Res. Cardiol.* 114:47. 10.3390/cells9010047 31673803 PMC9380897

[B201] Seidel T. Navankasattusas S. Ahmad A. Diakos N. A. Xu W. D. Tristani-Firouzi M. (2017a). Sheet-like remodeling of the transverse tubular system in human heart failure impairs excitation-contraction coupling and functional recovery by mechanical unloading. *Circulation* 135 1632–1645. 10.1161/circulationaha.116.024470 28073805 PMC5404964

[B202] Seidel T. Sankarankutty A. C. Sachse F. B. (2017b). Remodeling of the transverse tubular system after myocardial infarction in rabbit correlates with local fibrosis: a potential role of biomechanics. *Prog. Biophys. Mol. Biol.* 130 302–314. 10.1016/j.pbiomolbio.2017.07.006 28709857 PMC5716865

[B203] Severs N. J. Slade A. M. Powell T. Twist V. W. Jones G. E. (1985). Morphometric analysis of the isolated calcium-tolerant cardiac myocyte. Organelle volumes, sarcomere length, plasma membrane surface folds, and intramembrane particle density and distribution. *Cell Tissue Res.* 240 159–168. 3995538 10.1007/BF00217570

[B204] Shah S. J. Aistrup G. L. Gupta D. K. O’Toole M. J. Nahhas A. F. Schuster D. (2014). Ultrastructural and cellular basis for the development of abnormal myocardial mechanics during the transition from hypertension to heart failure. *Am. J. Physiol. Heart Circ. Physiol.* 306 H88–H100. 24186100 10.1152/ajpheart.00642.2013 PMC3920157

[B205] Sheard T. M. D. Hurley M. E. Colyer J. White E. Norman R. Pervolaraki E. (2019). Three-dimensional and chemical mapping of intracellular signaling nanodomains in health and disease with enhanced expansion microscopy. *ACS Nano* 13 2143–2157. 30715853 10.1021/acsnano.8b08742 PMC6396323

[B206] Sheldon C. A. Friedman W. F. Sybers H. D. (1976). Scanning electron microscopy of fetal and neonatal lamb cardiac cells. *J. Mol. Cell. Cardiol.* 8 853–862. 10.1016/0022-2828(76)90068-7 1003491

[B207] Shen X. van den Brink J. Hou Y. Colli D. Le C. Kolstad T. R. (2019). 3D dSTORM imaging reveals novel detail of ryanodine receptor localization in rat cardiac myocytes. *J. Physiol.* 597 399–418. 10.1113/jp277360 30412283 PMC6332759

[B208] Shiferaw Y. Aistrup G. L. Louch W. E. Wasserstrom J. A. (2020). Remodeling promotes proarrhythmic disruption of calcium homeostasis in failing atrial myocytes. *Biophys. J.* 118 476–491. 10.1016/j.bpj.2019.12.012 31889516 PMC6976802

[B209] Silbernagel N. Korner A. Balitzki J. Jaggy M. Bertels S. Richter B. (2020). Shaping the heart: structural and functional maturation of iPSC-cardiomyocytes in 3D-micro-scaffolds. *Biomaterials* 227:119551. 10.1016/j.biomaterials.2019.119551 31670034

[B210] Simpson F. O. (1965). The transverse tubular system in mammalian myocardial cells. *Am. J. Anat.* 117 1–17. 10.1002/aja.1001170102 14345833

[B211] Singh J. K. Barsegyan V. Bassi N. Marszalec W. Tai S. Mothkur S. (2017). T-tubule remodeling and increased heterogeneity of calcium release during the progression to heart failure in intact rat ventricle. *Physiol. Rep.* 5:e13540. 10.14814/phy2.13540 29279414 PMC5742703

[B212] Sipido K. R. Maes M. Van de Werf F. (1997). Low efficiency of Ca^2+^ entry through the Na(+)-Ca^2+^ exchanger as trigger for Ca^2+^ release from the sarcoplasmic reticulum. A comparison between L-type Ca^2+^ current and reverse-mode Na(+)-Ca^2+^ exchange. *Circ. Res.* 81 1034–1044. 10.1161/01.res.81.6.1034 9400385

[B213] Smyrnias I. Mair W. Harzheim D. Walker S. A. Roderick H. L. Bootman M. D. (2010). Comparison of the T-tubule system in adult rat ventricular and atrial myocytes, and its role in excitation-contraction coupling and inotropic stimulation. *Cell Calcium* 47 210–223. 10.1016/j.ceca.2009.10.001 20106523

[B214] Snopko R. M. Ramos-Franco J. Di Maio A. Karko K. L. Manley C. Piedras-Renteria E. (2008). Ca^2+^ sparks and cellular distribution of ryanodine receptors in developing cardiomyocytes from rat. *J. Mol. Cell. Cardiol.* 44 1032–1044. 10.1016/j.yjmcc.2008.03.015 18468619

[B215] Sobie E. A. Guatimosim S. Gomez-Viquez L. Song L. S. Hartmann H. Saleet Jafri M. (2006). The Ca^2+^ leak paradox and rogue ryanodine receptors: SR Ca 2+ efflux theory and practice. *Prog. Biophys. Mol. Biol.* 90 172–185. 10.1016/j.pbiomolbio.2005.06.010 16326215 PMC1484520

[B216] Soeller C. Cannell M. B. (1999). Examination of the transverse tubular system in living cardiac rat myocytes by 2-photon microscopy and digital image-processing techniques. *Circ. Res.* 84 266–275. 10.1161/01.res.84.3.266 10024300

[B217] Song L. S. Sobie E. A. McCulle S. Lederer W. J. Balke C. W. Cheng H. (2006). Orphaned ryanodine receptors in the failing heart. *Proc. Natl. Acad. Sci. U.S.A.* 103 4305–4310. 10.1073/pnas.0509324103 16537526 PMC1449688

[B218] Song Z. Liu M. B. Qu Z. (2018). Transverse tubular network structures in the genesis of intracellular calcium alternans and triggered activity in cardiac cells. *J. Mol. Cell. Cardiol.* 114 288–299. 10.1016/j.yjmcc.2017.12.003 29217432 PMC5801147

[B219] Sperelakis N. Rubio R. (1971). An orderly lattice of axial tubules which interconnect adjacent transverse tubules in guinea-pig ventricular myocardium. *J. Mol. Cell. Cardiol.* 2 211–220. 10.1016/0022-2828(71)90054-x 5117216

[B220] Spinozzi S. Liu C. Chen Z. Feng W. Zhang L. Ouyang K. (2020). Nexilin is necessary for maintaining the transverse-axial tubular system in adult cardiomyocytes. *Circ. Heart Fail.* 13:e006935. 10.1161/CIRCHEARTFAILURE.120.006935 PMC7583668 32635769

[B221] Stewart J. M. Page E. (1978). Improved stereological techniques for studying myocardial cell growth: application to external sarcolemma, T system, and intercalated disks of rabbit and rat hearts. *J. Ultrastruct. Res.* 65 119–134. 10.1016/s0022-5320(78)90050-3 366168

[B222] Stolen T. O. Hoydal M. A. Kemi O. J. Catalucci D. Ceci M. Aasum E. (2009). Interval training normalizes cardiomyocyte function, diastolic Ca^2+^ control, and SR Ca^2+^ release synchronicity in a mouse model of diabetic cardiomyopathy. *Circ. Res.* 105 527–536. 10.1161/circresaha.109.199810 19679837

[B223] Sun X. H. Protasi F. Takahashi M. Takeshima H. Ferguson D. G. Franzini-Armstrong C. (1995). Molecular architecture of membranes involved in excitation-contraction coupling of cardiac muscle. *J. Cell Biol.* 129 659–671. 10.1083/jcb.129.3.659 7730402 PMC2120446

[B224] Swift F. Birkeland J. A. Tovsrud N. Enger U. H. Aronsen J. M. Louch W. E. (2008). Altered Na^+^/Ca^2+^ -exchanger activity due to downregulation of Na^+^/K^+^-ATPase alpha_2_-isoform in heart failure. *Cardiovasc. Res.* 78 71–78. 10.1093/cvr/cvn013 18203708

[B225] Swift F. Franzini-Armstrong C. Oyehaug L. Enger U. H. Andersson K. B. Christensen G. (2012). Extreme sarcoplasmic reticulum volume loss and compensatory T-tubule remodeling after Serca2 knockout. *Proc. Natl. Acad. Sci. U.S.A.* 109 3997–4001. 10.1073/pnas.1120172109 22355118 PMC3309775

[B226] Swift F. Stromme T. A. Amundsen B. Sejersted O. M. Sjaastad I. (2006). Slow diffusion of K^+^ in the T tubules of rat cardiomyocytes. *J. Appl. Physiol. (1985)* 101 1170–1176. 10.1152/japplphysiol.00297.2006 16763106

[B227] Takeshima H. Komazaki S. Nishi M. Iino M. Kangawa K. (2000). Junctophilins: a novel family of junctional membrane complex proteins. *Mol. Cell* 6 11–22. 10.1016/s1097-2765(00)00003-4 10949023

[B228] Tarzia V. Di Giammarco G. Di Mauro M. Bortolussi G. Maccherini M. Tursi V. (2016). From bench to bedside: can the improvements in left ventricular assist device design mitigate adverse events and increase survival? *J. Thorac. Cardiovasc. Surg.* 151 213–217. 10.1016/j.jtcvs.2015.09.107 26548997

[B229] Tasfaout H. Buono S. Guo S. Kretz C. Messaddeq N. Booten S. (2017). Antisense oligonucleotide-mediated Dnm2 knockdown prevents and reverts myotubular myopathy in mice. *Nat. Commun.* 8:15661. 10.1038/ncomms15661 PMC5467247 28589938

[B230] Thomas M. J. Sjaastad I. Andersen K. Helm P. J. Wasserstrom J. A. Sejersted O. M. (2003). Localization and function of the Na^+^/Ca^2+^ -exchanger in normal and detubulated rat cardiomyocytes. *J. Mol. Cell. Cardiol.* 35 1325–1337. 10.1016/j.yjmcc.2003.08.005 14596789

[B231] Tidball J. G. Cederdahl J. E. Bers D. M. (1991). Quantitative analysis of regional variability in the distribution of transverse tubules in rabbit myocardium. *Cell Tissue Res.* 264 293–298. 10.1007/bf00313966 1715241

[B232] Uchida K. Lopatin A. N. (2018). Diffusional and electrical properties of T-Tubules are governed by their constrictions and dilations. *Biophys. J.* 114 437–449. 10.1016/j.bpj.2017.11.3742 29401441 PMC5984979

[B233] Vaidya Y. Dhamoon A. S. (2019). *StatPearls.* Treasure Island (FL): StatPearls Publishing StatPearls Publishing LLC.

[B234] van Oort R. J. Garbino A. Wang W. Dixit S. S. Landstrom A. P. Gaur N. (2011). Disrupted junctional membrane complexes and hyperactive ryanodine receptors after acute junctophilin knockdown in mice. *Circulation* 123 979–988. 10.1161/circulationaha.110.006437 21339484 PMC3056402

[B235] Wagner E. Lauterbach M. A. Kohl T. Westphal V. Williams G. S. Steinbrecher J. H. (2012). Stimulated emission depletion live-cell super-resolution imaging shows proliferative remodeling of T-tubule membrane structures after myocardial infarction. *Circ. Res.* 111 402–414. 22723297 10.1161/CIRCRESAHA.112.274530 PMC4219578

[B236] Wakili R. Yeh Y. H. Yan Qi X. Greiser M. Chartier D. Nishida K. (2010). Multiple potential molecular contributors to atrial hypocontractility caused by atrial tachycardia remodeling in dogs. *Circ. Arrhythm. Electrophysiol.* 3 530–541. 20660541 10.1161/CIRCEP.109.933036

[B237] Wang H. Li Z. Wang J. Sun K. Cui Q. Song L. (2010). Mutations in NEXN, a Z-disc gene, are associated with hypertrophic cardiomyopathy. *Am. J. Hum. Genet.* 87 687–693. 20970104 10.1016/j.ajhg.2010.10.002 PMC2978958

[B238] Wang W. Landstrom A. P. Wang Q. Munro M. L. Beavers D. Ackerman M. J. (2014). Reduced junctional Na^+^/Ca^2+^ -exchanger activity contributes to sarcoplasmic reticulum Ca^2+^ leak in junctophilin-2-deficient mice. *Am. J. Physiol. Heart Circ. Physiol.* 307 H1317–H1326. 25193470 10.1152/ajpheart.00413.2014 PMC4217007

[B239] Wang X. Xie W. Zhang Y. Lin P. Han L. Han P. (2010). Cardioprotection of ischemia/reperfusion injury by cholesterol-dependent MG53-mediated membrane repair. *Circ. Res.* 107 76–83. 20466981 10.1161/CIRCRESAHA.109.215822

[B240] Wang Y. Chen B. Huang C. K. Guo A. Wu J. Zhang X. (2018). Targeting calpain for heart failure therapy: implications from multiple murine models. *JACC Basic Transl. Sci.* 3 503–517. 30175274 10.1016/j.jacbts.2018.05.004 PMC6115647

[B241] Ward M. L. Crossman D. J. (2014). Mechanisms underlying the impaired contractility of diabetic cardiomyopathy. *World J. Cardiol.* 6 577–584. 10.4330/wjc.v6.i7.577 25068018 PMC4110606

[B242] Wei S. Guo A. Chen B. Kutschke W. Xie Y. P. Zimmerman K. (2010). T-tubule remodeling during transition from hypertrophy to heart failure. *Circ. Res.* 107 520–531. 10.1161/circresaha.109.212324 20576937 PMC2927862

[B243] Wright P. T. Bhogal N. K. Diakonov I. Pannell L. M. K. Perera R. K. Bork N. I. (2018). Cardiomyocyte membrane structure and cAMP compartmentation produce anatomical variation in beta2AR-cAMP responsiveness in murine hearts. *Cell Rep.* 23 459–469. 10.1016/j.celrep.2018.03.053 29642004 PMC5912947

[B244] Wu C. Y. Chen B. Jiang Y. P. Jia Z. Martin D. W. Liu S. (2014). Calpain-dependent cleavage of junctophilin-2 and T-tubule remodeling in a mouse model of reversible heart failure. *J. Am. Heart Assoc.* 3: e000527. 10.1161/JAHA.113.000527 PMC4309042 24958777

[B245] Wu C. Y. Jia Z. Wang W. Ballou L. M. Jiang Y. P. Chen B. (2011). PI3Ks maintain the structural integrity of T-tubules in cardiac myocytes. *PLoS One* 6:e24404. 10.1371/journal.pone.0024404 21912691 PMC3166327

[B246] Wu H. D. Xu M. Li R. C. Guo L. Lai Y. S. Xu S. M. (2012). Ultrastructural remodelling of Ca^2+^ signalling apparatus in failing heart cells. *Cardiovasc. Res.* 95 430–438. 10.1093/cvr/cvs195 22707157 PMC3422078

[B247] Xie Y. P. Chen B. Sanders P. Guo A. Li Y. Zimmerman K. (2012). Sildenafil prevents and reverses transverse-tubule remodeling and Ca^2+^ handling dysfunction in right ventricle failure induced by pulmonary artery hypertension. *Hypertension* 59 355–362. 10.1161/hypertensionaha.111.180968 22203744 PMC3266850

[B248] Xie Y. Yang Y. Galice S. Bers D. M. Sato D. (2019). Size matters: ryanodine receptor cluster size heterogeneity potentiates calcium waves. *Biophys. J.* 116 530–539. 10.1016/j.bpj.2018.12.017 30686487 PMC6369574

[B249] Xu M. Wu H. D. Li R. C. Zhang H. B. Wang M. Tao J. (2012). Mir-24 regulates junctophilin-2 expression in cardiomyocytes. *Circ. Res.* 111 837–841. 10.1161/circresaha.112.277418 22891046 PMC3611051

[B250] Xu M. Zhou P. Xu S. M. Liu Y. Feng X. Bai S. H. (2007). Intermolecular failure of L-type Ca^2+^ channel and ryanodine receptor signaling in hypertrophy. *PLoS Biol* 5:e21. 10.1371/journal.pbio.0050021 17214508 PMC1764437

[B251] Yamakawa S. Wu D. Dasgupta M. Pedamallu H. Gupta B. Modi R. (2021). Role of t-tubule remodeling on mechanisms of abnormal calcium release during heart failure development in canine ventricle. *Am. J. Physiol. Heart Circ. Physiol.* 320 H1658–H1669. 33635163 10.1152/ajpheart.00946.2020 PMC8260383

[B252] Zhang C. Chen B. Guo A. Zhu Y. Miller J. D. Gao S. (2014). Microtubule-mediated defects in junctophilin-2 trafficking contribute to myocyte transverse-tubule remodeling and Ca^2+^ handling dysfunction in heart failure. *Circulation* 129 1742–1750. 10.1161/circulationaha.113.008452 24519927 PMC4006305

[B253] Zhang C. Chen B. Wang Y. Guo A. Tang Y. Khataei T. (2017). MG53 is dispensable for T-tubule maturation but critical for maintaining T-tubule integrity following cardiac stress. *J. Mol. Cell. Cardiol.* 112 123–130. 10.1016/j.yjmcc.2017.08.007 28822805 PMC5682927

[B254] Ziman A. P. Gomez-Viquez N. L. Bloch R. J. Lederer W. J. (2010). Excitation-contraction coupling changes during postnatal cardiac development. *J. Mol. Cell. Cardiol.* 48 379–386. 10.1016/j.yjmcc.2009.09.016 19818794 PMC3097073

