# Corrigendum: The Physiology and Pathophysiology of T-Tubules in the Heart

**DOI:** 10.3389/fphys.2021.790227

**Published:** 2021-10-26

**Authors:** Ingunn E. Setterberg, Christopher Le, Michael Frisk, Harmonie Perdreau-Dahl, Jia Li, William E. Louch

**Affiliations:** ^1^Institute for Experimental Medical Research, Oslo University Hospital and University of Oslo, Oslo, Norway; ^2^KG Jebsen Centre for Cardiac Research, University of Oslo, Oslo, Norway

**Keywords:** t-tubules, dyad, cardiomyocyte, calcium homeostasis, heart failure

**Harmonie Perdreau-Dahl** was not included as an author in the published article. The corrected Author Contributions Statement appears below.

## Author Contributions

All authors contributed to the writing of the article and development of the figures.

The authors apologize for this error and state that this does not change the scientific conclusions of the article in any way. The original article has been updated.

## Publisher's Note

All claims expressed in this article are solely those of the authors and do not necessarily represent those of their affiliated organizations, or those of the publisher, the editors and the reviewers. Any product that may be evaluated in this article, or claim that may be made by its manufacturer, is not guaranteed or endorsed by the publisher.

